# Supplementary material for: Small molecule inhibitors of RAS-effector protein interactions derived using an intracellular antibody fragment
Source: Nat Commun. 2018 Aug 9;9:3169. doi: 10.1038/s41467-018-05707-2 (PMC6085350; doi:10.1038/s41467-018-05707-2)
Supplement: Supplementary file 1 — Supplementary Information [file 41467_2018_5707_MOESM1_ESM.pdf]

Small molecule inhibitors of RAS-effector protein interactions derived using an intracellular antibody fragment

Camilo E. Quevedo, et al

Supplementary Information

Supplementary Note 1

General procedure for the synthesis of the (2,3-dihydrobenzo[b][1,4]dioxin-2-yl)methanamine intermediates.

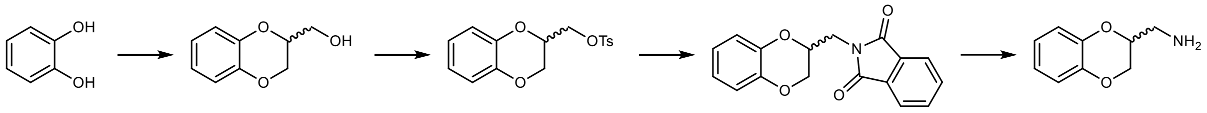

The requisite amines were prepared using a modification of a literature procedure [59,60]. The stereoselectivity was implemented in the first step using the appropriate epichlorohydrin derivative. Potassium carbonate (1.45 g, 10.5 mmol) was added to a solution of catechol (1.17 g, 10.5 mmol) and the requisite epichlorohydrin derivative (2.00 g, 8.76 mmol) in DMF (20 mL) and the mixture was stirred at 60 °C for 24 h. The mixture was cooled down, diluted with ice-water, extracted with Et_2O (100 mL), and washed multiple times with water/brine (1:1) to remove the DMF. The organic phase was dried (Na_2SO_4), filtered and concentrated *in vacuo* to afford the desired product as a white solid that did not require further purification.
Using *p*-toluenesulfonic acid, (2*R*)-(−)-glycidyl ester, (*S*)-(2,3-dihydrobenzo[*b*][1,4]dioxin-2-yl)methanol was obtained as an off-white solid (1.42 g, 97%).
Using *p*-toluenesulfonic acid, (2*S*)-(−)-glycidyl ester, (*R*)-(2,3-dihydrobenzo[*b*][1,4]dioxin-2-yl)methanol was obtained as an off-white solid (1.44 g, 99%).
Using epichlorohydrin, (*rac*)-(2,3-dihydrobenzo[*b*][1,4]dioxin-2-yl)methanol was obtained as an off-white solid (1.43 g, 98%). The data were consistent with those of the literature.
p-Toluenesulfonyl chloride (345 mg, 1.81 mmol) was added to a solution of the alcohol (300 mg, 1.81 mmol) in pyridine (5 mL) and the mixture stirred at room temperature for 18 h. Et_2O (20 mL) was then added and the organic phase was washed with HCl (1N, aq.) until neutral, then with water and brine. The organic phase was dried (Na_2SO_4), filtered and concentrated *in vacuo* to obtain the crude compound as a yellow oil that was purified *via* column on silica gel (eluent pentane:EtOAc (4:1) to afford the desired compound as a pale yellow solid (545 mg, 95%).
Potassium phthalimide (278 mg, 1.50 mmol) was added to a solution of the tosylate (400 mg, 1.25 mmol) in DMF (5 mL). The mixture was stirred at 150 °C for 1 h, cooled to room temperature, and poured to ice-water whilst vigorously stirring. The suspension was stirred for 30 min and the resulting solid filtered, washed with water, NaOH (2M, aq.) then water and dried *in vacuo* to give the desired product as a white solid (325 mg, 88%).
To a solution of the phthalimide derivative (247 mg, 0.837 mmol) in EtOH (10 mL) was added hydrazine hydrate (84 µL, 1.09 mmol) and the mixture was stirred at reflux for 2 h. After cooling, HCl (1N, aq.) was added until pH 1 and the reaction was stirred for 15 min. The formed white solid was filtered and washed with EtOH. The filtrate was concentrated *in vacuo*, and the residue partitioned between Et_2O and NaOH (0.5 M, aq.). The aqueous phase was extracted further with Et_2O, dried (Na_2SO_4) filtered and concentrated *in vacuo* to obtain the (2,3-dihydrobenzo[b][1,4]dioxin-2-yl)methanamine intermediate as a colourless oil (110 mg, 80%).
^1H NMR (400 MHz, $CDCl_3$) δ = 6.86 (m, 4H), 4.27 (dd, J = 2.4, 11.4 Hz, 1H), 4.13 (m, 1H), 4.02 (dd, J = 7.8, 11.2 Hz, 1H), 2.99 (dd, J = 3.2, 5.6 Hz, 2H); MS (ESI+) 166.1 $(M+H)^+$.

Supplementary Note 2

General procedure for the preparation of Abd-2, Abd-2a and Abd-2b

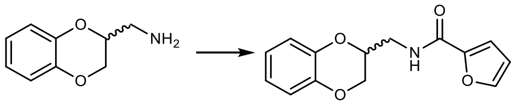

To a solution of the requisite amine (100 mg, 0.605 mmol) in CH_2Cl_2 (5 mL) was sequentially added Et_3N (505 µL, 3.63 mmol), followed by 2-furoyl chloride (60 µL, 0.605 mmol). The resulting reaction was stirred for 16 h at room temperature before addition of water (10 mL). The aqueous phase was extracted with CH_2Cl_2 (20 mL), the combined organic phase washed with brine (25 mL), dried (Na_2SO_4), filtered and concentrated *in vacuo*. The resulting brown oil was purified *via* column on silica gel (eluent pentane:acetone, 4:1). Following the general procedure, **Abd-2** was obtained as a white solid (151 mg, 96%), $[\alpha]_D$ = 0 (c = 1.02, $CHCl_3$); compound **Abd-2a** was obtained as a yellow oil (149 mg, 96%), $[\alpha]_D$ =+21.3 (c = 1.07, $CHCl_3$);. compound **Abd-2b** was obtained as a yellow oil (152 mg, 96%); $[\alpha]_D$ =–17.8 (c = 1.05, $CHCl_3$); the enantiomers and racemic compounds had the same NMR and MS data; ^{1}H NMR (400 MHz, DMSO-d_6) δ = 8.63 (t, *J* 5.8 Hz, 1H), 7.86 (dd *J* 1.8, 0.8 Hz, 1H), 7.15 (dd, *J* 3.4, 0.8 Hz, 1H), 6.88-6.81 (m, 4H), 6.63 (dd, *J* 3.4, 1.8 Hz, 1H), 4.42-4.29 (2H, m), 3.96 (dd, *J* 11.8, 7.4 Hz, 1H), 3.48 (ddt, *J* 33.6, 13.9, 6.1 Hz, 2H). ^{13}C NMR (75 MHz, DMSO-d_6) δ = 158.2, 147.6, 145.2, 143.0, 142.8, 121.5, 121.3, 117.2, 117.0, 113.8, 111.9, 71.6, 65.6; MS (ESI+) 260.1 $(M+H)^+$; HRMS (ESI^+) $[C_{14}H_{14}NO_4]$ requires 260.0923, found 260.927; LCMS Rt: 2.26 min, m/z 260.1 $[M+H]^+$.

Supplementary Note 3

Synthesis of Abd-3:

(6-Chloro-2,3-dihydrobenzo[b][1,4]dioxin-2-yl)methanamine (Abd-3)

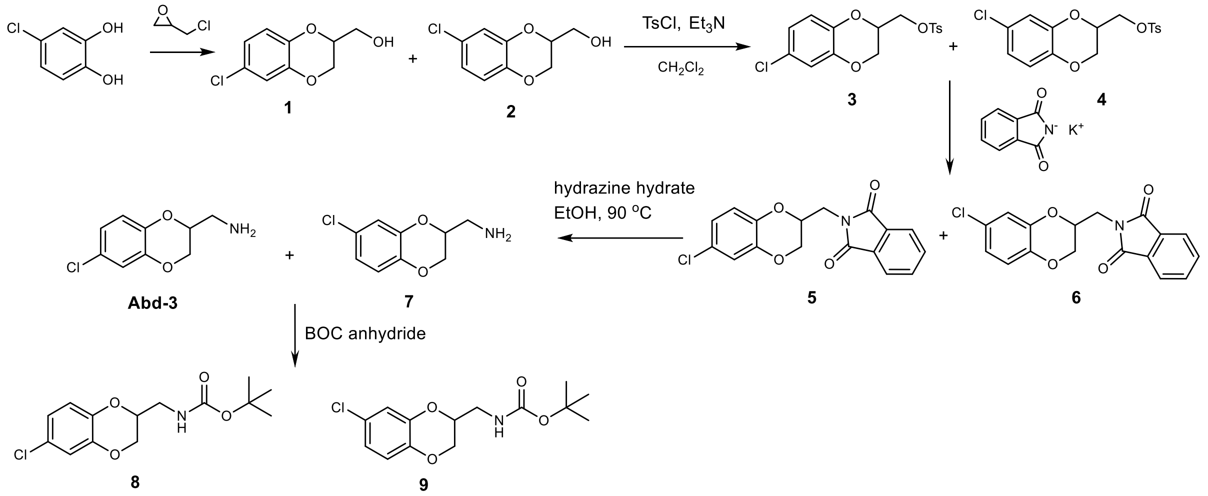

Amine protected intermediate was prepared following an amended literature procedure using a four-step synthesis[59]. To a solution of 4-chlorocatechol (21.2 g, 147 mmol) in DMF (210 mL) under nitrogen atmosphere was added K_2CO_3 (30.3 g, 220 mmol) followed by gradual addition of (chloromethyl)cyclopropane (20.3 g, 220 mmol) at room temperature. The mixture was stirred at 90°C for 18 h. The reaction was diluted with water (500 mL) and extracted with EtOAc (2 x 200 mL). The combined organic layer was washed with water (300 mL) and brine (300 mL), dried (Na_2SO_4) and concentrated *in vacuo*. The crude product was purified by column chromatography on silica (20-30% EtOAc/pet ether) to afford a mixture of 6-chloro- and 7-chloro-2,3-dihydro-benzo[1,4]dioxin-2-yl)-methanol (**1** and **2**) as a pale yellow solid (26.0 g, 90%). To a solution of 6-chloro- and 7-chloro-2,3-dihydro-benzo[1,4]dioxin-2-yl)-methanol **1** and **2** (26.0 g, 130 mmol) in CH_2Cl_2 (520 mL) under nitrogen atmosphere, was sequentially added Et_3N (53.6 mL, 389 mmol) dropwise over 10 min, and TsCl (27.2 g, 143 mmol) portion-wise over 30 min. The resulting mixture was stirred at room temperature for 18 h, quenched with water, and extracted with CH_2Cl_2 (2 x 300 mL). The combined organic extracts were washed with water (2 x 200 mL) and brine (2 x 200 mL), dried (Na_2SO_4) and concentrated *in vacuo*. The crude product was purified by column chromatography on silica gel (20-30% EtOAc:pet ether) to afford a mixture of toluene-4-sulfonic acid 6-chloro-2,3-dihydro-benzo[1,4]dioxin-2-ylmethyl ester and toluene-4-sulfonic acid 7-chloro-2,3-dihydro-benzo[1,4]dioxin-2-ylmethyl ester (40.0 g, 87%) **3** and **4** as an off-white solid.

Potassium phthalimide (33.5 g, 181 mmol) was added to a solution of the tosylate derivatives **3** and **4** (40.0 g, 113 mmol) in DMF (400 mL) under nitrogen atmosphere. The reaction was stirred at 90 °C for 3 h. The mixture was quenched with water and extracted with EtOAc (3 x 300 mL). The combined organic layers were washed with water (2 x 300 mL), brine (2 x 300 mL), dried (Na_2SO_4) and concentrated *in vacuo*. The crude product was purified by column chromatography on silica gel (20-30% EtOAc:pet ether) to afford a mixture of 2-(6-chloro-2,3-dihydro-benzo[1,4]dioxin-2-ylmethyl)-isoindole-1,3-dione and 2-(7-chloro-2,3-dihydro-benzo[1,4]dioxin-2-ylmethyl)-isoindole-1,3-dione (32.4 g, 87%) **5** and **6** as an off-white solid.

Hydrazine hydrate (49.2 g, 985 mmol) was added to a solution of 2-(6-chloro-2,3-dihydro-benzo[1,4]dioxin-2-ylmethyl)-isoindole-1,3-dione and 2-(7-chloro-2,3-dihydro-benzo[1,4]dioxin-2-ylmethyl)-isoindole-1,3-dione (32.4 g, 98.5 mmol) **5** and **6** in EtOH (390 mL) under nitrogen atmosphere, and the resulting mixture stirred at 90 °C for 2 h. The reaction was cooled down, filtered and the residue was washed with CH_2Cl_2 (2 x 200 mL). The combined filtrates were concentrated *in vacuo* to afford a mixture of (6-chloro-2,3-

dihydro-benzo[1,4]dioxin-2-yl)-methylamine **Abd-3** and (7-chloro-2,3-dihydro-benzo[1,4]dioxin-2-yl)-methylamine (18.5 g, 94%) **7** as a pale yellow liquid.

The resulting mixture of (6-chloro-2,3-dihydro-benzo[1,4]dioxin-2-yl)-methylamine and (7-chloro-2,3-dihydro-benzo[1,4]dioxin-2-yl)-methylamine **Abd-3** and **7** (18.3 g, 91.2 mmol) was dissolved in 1:1 THF:water (184 mL), and $NaHCO_3$ (22.9 g, 274 mmol) was added, followed by portion-wise addition of Boc_2O (21.9 g, 100 mmol). The reaction was stirred for 16 h, extracted with EtOAc (3 x 200 mL) and the combined organic extracts were dried (Na_2SO_4) and concentrated *in vacuo*. The crude product was purified by column chromatography on silica (5-10% EtOAc:pet ether) to afford a mixture of (6-chloro-2,3-dihydro-benzo[1,4]dioxin-2-ylmethyl)-carbamic acid *tert*-butyl ester and (7-chloro-2,3-dihydro-benzo[1,4]dioxin-2-ylmethyl)-carbamic acid *tert*-butyl ester (21.3 g, 78%) **8** and **9** as an off-white solid.

A 10 gram sample of the resulting mixture was purified by supercritical fluid chromatography (Lux Amylose-2, 250 x 30mm) to afford **(6-chloro-2,3-dihydro-benzo[1,4]dioxin-2-ylmethyl)-carbamic acid *tert*-butyl ester 8** (4.5 g) AnalpH2_MeCN_UPLC_4min; Rt: 2.35 min, m/z 244.1 [M+H]+; AnalpH2_MeCN_UPLC_6min: Rt: 2.12 min. and **(7-chloro-2,3-dihydro-benzo[1,4]dioxin-2-ylmethyl)-carbamic acid *tert*-butyl ester 9** (1.5 g) AnalpH2_MeCN_UPLC_4min; Rt: 2.35 min, m/z 244.1 [M+H]+; AnalpH2_MeCN_UPLC_6min: Rt: 3.43 min.

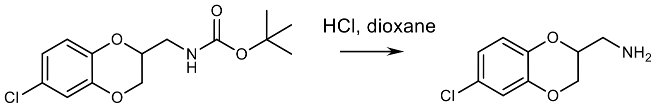

To a solution of (6-chloro-2,3-dihydro-benzo[1,4]dioxin-2-ylmethyl)-carbamic acid *tert*-butyl ester **8** (4.50 g, 15.0 mmol) in 1,4-dioxane (47 mL) at 0 °C was added HCl in 1,4-dioxane (1M, 47 mL) and the reaction mixture stirred for 18 h at room temperature. The solution was concentrated *in vacuo* and treated with $NaHCO_3$ (aq. sat. sol.), then extracted with 10% MeOH in CH_2Cl_2 (3 x 50 mL). The combined organic extracts were dried (Na_2SO_4) and concentrated *in vacuo* to afford (6-chloro-2,3-dihydro-benzo[1,4]dioxin-2-yl)-methylamine **Abd-3** (2.80 g, 94%) as an off-white solid.

^{1}H NMR (400 MHz, $CDCl_3$) δ = 6.88 (d, *J* 1.2 Hz, 1H), 6.81 (s, 2H), 4.27 (d, *J* 10.8 Hz, 1H), 4.12 (dd, *J* 13.0, 6.8 Hz, 1H), 2.95–2.98 (m, 2H), 4.00 (dd, *J* 11.4, 7.4 Hz, 1H), 1.25 (br s, 2H),; AnalpH2_MeCN_UPLC_4min; Rt: 1.17 min, m/z 200.1 [M+H]+

Supplementary Note 4

Synthesis of Abd-4:

***N*-((2,3-dihydrobenzo[b][1,4]dioxin-2-yl)methyl)-4(2(dimethylamino)ethoxy) benzamide (Abd-4)**

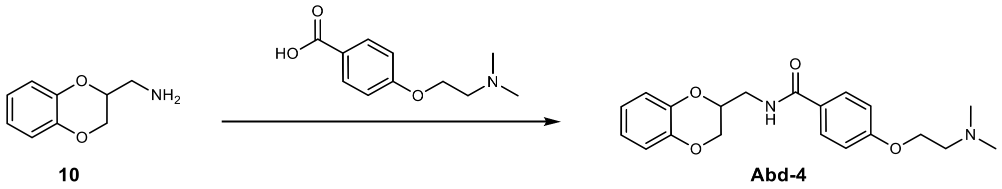

To a stirred solution of 4-(2-dimethylamino-ethoxy)-benzoic acid (112 mg, 0.530 mmol) in DMF (2 mL) was added Et_3N (217 µL, 1.61 mmol) and HATU (264 mg, 0.700 mmol) and the reaction stirred at room temperature for 5 min. 2,3-Dihydro-benzo[1,4]dioxin-2-yl)-methylamine **10** (88 mg, 0.540 mmol) was then added and the mixture stirred at room temperature for 72 h. The reaction was concentrated *in vacuo* and the resulting residue dissolved in MeOH and passed through an SCX-2 cartridge eluting with 2M NH_3 in MeOH to afford the crude product. The compound was purified by reverse phase preparative HPLC to afford *N*-(2,3-dihydro-benzo[1,4]dioxin-2-ylmethyl)-4-(2-dimethylamino-ethoxy)-benzamide **Abd-4** (69 mg, 36%) as a white solid. AnalpH2_MeOH_QC_V1: Rt: 4.73 min, m/z 357.2 $[M+H]^+$; AnalpH9_MeOH_QC_V1: Rt: 7.68 min, m/z 357.2 $[M+H]^+$; m.p. (MeOH) 104-106 °C; ^1H NMR (600 MHz, CD_3OD) δ 7.81 (d, *J* 8.8 Hz, 2H), 7.02 (d, *J* 8.8 Hz, 2H), 6.88-6.85 (m, 1H), 6.84-6.78 (m, 3H), 4.37-4.34 (m, 1H), 4.31 (dd, *J* 11.6, 2.4 Hz, 1H), 4.17 (t, *J* 5.5 Hz, 2H), 3.98 (dd, *J* 11.4, 7.0 Hz, 1H), 3.66 (qd, *J* 13.9, 9.9 Hz, 2H), 2.79 (t, *J* 5.5 Hz, 2H), 2.35 (s, 6H), NH was not observed; ^{13}C NMR (150 MHz, CD_3OD) δ 170.4, 163.2, 144.8, 144.6, 130.4, 127.8, 122.7, 122.5, 118.5, 118.2, 115.5, 73.5, 67.4, 67.0, 59.1, 46.0, 41.5; *m/z* (ESI^+) 358 ($[M+H]^+$); HRMS (ESI^+) $[C_{20}H_{25}N_2O_4]$ requires 357.18143, found 357.18157.

Supplementary Note 5

Synthesis of Abd-5:

(8-bromo-2,3-dihydrobenzo[b][1,4]dioxin-2-yl)methyl 4-methylbenzenesulfonate (12)

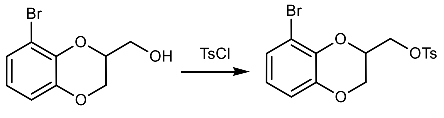

11 **12**

To a solution of (8-bromo-2,3-dihydro-benzo[1,4]dioxin-2-yl)-methanol **11** (40.0 g, 163 mmol) in CH_2Cl_2 (400 mL) was added Et_3N (89 mL, 655 mmol) dropwise over 10 min, then TsCl (37.4 g, 196 mmol) was added portion-wise over 10 min. The reaction mixture was stirred at room temperature for 3 h, quenched with water, extracted with CH_2Cl_2 (2 x 100 mL). The combined organic layers were washed with water (2 x 200 mL) and brine (2 x 200 mL), dried (Na_2SO_4) and concentrated *in vacuo* to afford the crude product, which was purified by column chromatography on silica gel (10% EtOAc:pet ether) to afford (8-bromo-2,3-dihydrobenzo[b][1,4]dioxin-2-yl)methyl 4-methylbenzenesulfonate **12** (20.0 g, 25%) as a yellow solid. AnalpH2_MeCN_UPLC_4min: Rt: 2.37 min, 399.1, 401.0 $[M+H]^+$; ^1H NMR ($CDCl_3$, 400 MHz) 7.79-7.83 (m, 1H), 7.35 (d, *J* 8.4 Hz, 2H), 7.08 (d, *J* 7.6 Hz, 1H), 6.83-6.77 (m, 1H), 6.71 (t, *J* 8.2 Hz, 1H), 4.51-4.48 (m, 1 H), 4.30-4.17 (m, 3H), 4.10-4.02 (m, 1H), 2.45 (s, 3H).

2-((8-bromo-2,3-dihydrobenzo[b][1,4]dioxin-2-yl)methyl)isoindoline-1,3-dione (14)

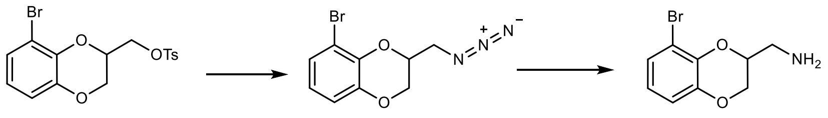

12 **13** **14**

To a solution of (8-bromo-2,3-dihydrobenzo[b][1,4]dioxin-2-yl)methyl 4-methylbenzene sulfonate **12** (20.0 g, 50.1 mmol) in DMF (200 mL) was added NaN_3 (32.5 g, 501 mmol) at room temperature. The mixture was stirred at 70 °C under N_2 for 2 h, cooled down, treated with water and extracted with EtOAc (3 x 300 mL). The combined organic layer was washed with water (2 x 200 mL) and brine (2 x 200 mL), dried (Na_2SO_4) and concentrated *in vacuo* to afford 2-azidomethyl-8-bromo-2,3-dihydro-benzo[1,4]dioxine (**13**), which was used directly in the next step without any further purification.
To a solution of 2-azidomethyl-8-bromo-2,3-dihydro-benzo[1,4]dioxine **13** (9.00 g, 33.3 mmol) in THF (100 mL) and water (10 mL) was added triphenylphosphine (9.60 g, 36.6 mmol). The mixture was stirred at 50 °C for 1 h, and then the reaction mixture was concentrated *in vacuo*. The crude compound was purified by column chromatography on silica gel (20% MeOH:CH_2Cl_2) to afford (8-bromo-2,3-dihydro-benzo[1,4]dioxin-2-yl)-methylamine **14** (5.50 g, 45% over 2 steps) as a yellow liquid.
AnalpH2_MeCN_UPLC_4min: Rt: 1.09 min, 244.1, 246.1 $[M+H]^+$.
1H NMR (DMSO, 400 MHz) 7.12-7.10 (m, 1H), 6.89 (dd, *J* 8.4, 1.2 Hz, 1H), 6.76 (t, *J* 8.2Hz, 1H), 4.38 (dd, *J* 11.8, 2.2 Hz, 1H), 4.17-4.11 (m, 1H), 4.02 (dd, *J* 11.6, 7.2Hz, 1H), 2.88–2.75 (m, 2H), 1.59 (br s, 1H).

***N*-((8-bromo-2,3-dihydrobenzo[b][1,4]dioxin-2-yl)methyl)-4-(2(dimethylamino) ethoxy) benzamide (15)**

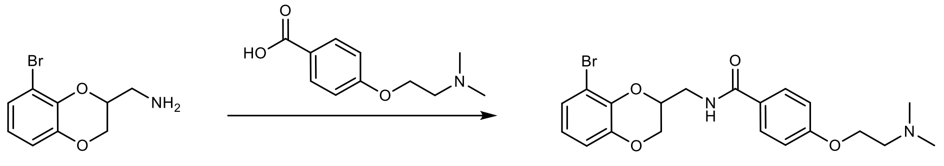

14 **15**

To a solution of **14** (100 mg, 0.411 mmol) in DMF (3 mL) was added HATU (219 mg, 0.574 mmol), *N*,*N*-diisopropylethylamine (286 µL, 1.64 mmol) and 4-(2-dimethylamino-ethoxy)-benzoic acid (103 mg, 0.494 mmol), and the reaction mixture was stirred at room temperature for 16 h. The reaction was diluted with EtOAc (10 mL) and washed with a 50/50 solution of water and brine (3 x 50 mL). The organic phase was dried (Na_2SO_4) and concentrated *in vacuo* the crude compound, which was purified by column chromatography on silica gel (10% MeOH:CH_2Cl_2) to afford *N*-((8-bromo-2,3-dihydrobenzo[b][1,4]dioxin-2-yl)methyl)-4-(2(dimethylamino)ethoxy)benzamide **15** (164 mg, 92%) as a pale orange glassy solid.

m.p. (MeOH) 143-145 °C; ^{1}H NMR (400 MHz, CD_3OD) δ 7.85 (d, *J* 8.6 Hz, 2H), 7.07 (dd, *J* 7.9, 1.3 Hz, 1H), 7.02 (d, *J* 8.8 Hz, 2H), 6.83 (dd, *J* 8.1, 1.5 Hz, 1H), 6.72 (t, *J* 8.1 Hz, 1H), 4.48-4.43 (m, 1H), 4.35 (dd, *J* 11.6, 2.1 Hz, 1H), 4.17 (t, *J* 5.4 Hz, 2H), 4.02 (dd, *J* 11.6, 6.7 Hz, 1H), 3.73 (dd, *J* 13.9, 6.4 Hz, 1H), 3.65 (dd, *J* 13.9, 5.8 Hz, 1H), 2.84 (t, *J* 5.3 Hz, 2H), 2.38 (s, 6H), NH was not observed; ^{13}C NMR (150 MHz, CD_3OD) δ 170.3, 163.1, 145.8, 141.8, 130.5, 127.8, 126.4, 122.9, 117.6, 115.5, 111.9, 74.0, 67.3, 66.7, 59.0, 45.8, 41.2; *m/z* (ESI$^+$) 436 ([M+H]$^+$); HRMS (ESI$^+$) [$C_{20}H_{24}BrN_2O_4$] requires 437.0835, found 437.0832.

4-(2-Dimethylamino-ethoxy)-*N*-[8-(6-methoxy-pyridin-2-yl)-2,3-dihydro-benzo[1,4] dioxin-2-ylmethyl]-benzamide (Abd-5)

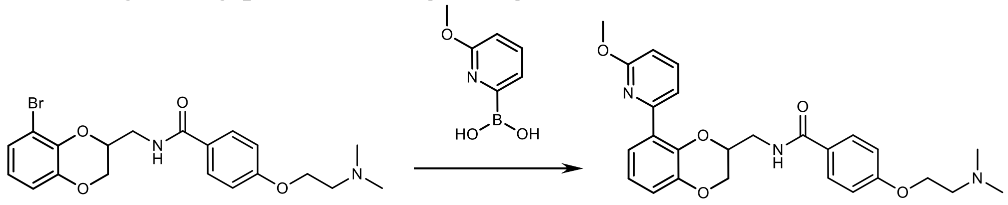

15 **Abd-5**

To a solution of *N*-(8-bromo-2,3-dihydro-benzo[1,4]dioxin-2-ylmethyl)-4-(2-dimethylamino-ethoxy)-benzamide **15** (135 mg, 0.310 mmol) in 1,4-dioxane:water (9:1, 10 mL) was added 6-methoxypyridine-2-boronic acid (95 mg, 0.62 mmol), $PdCl_2$(dtbpf) (20 mg, 0.031 mmol) and Na_2CO_3 (72 mg, 0.680 mmol). The resulting mixture was degassed with N_2 for 15 min and heated to 110 °C for 30 min. The reaction was allowed to cool and concentrated *in vacuo*, dissolved in MeOH, loaded onto a SCX-2 cartridge, washed with MeOH then eluted with 2M NH_3 in MeOH. The product-containing fractions were concentrated *in vacuo* to yield the crude material which was purified by reverse phase preparative HPLC to afford 4-(2-dimethylamino-ethoxy)-*N*-[8-(6-methoxy-pyridin-2-yl)-2,3-dihydro-benzo[1,4]dioxin-2-yl-methyl]-benzamide **Abd-5** (33 mg, 51%) as a white solid. AnalpH2_MeOH_QC_V1: Rt: 5.58 min, m/z 464.3 [M+H]+; AnalpH9_MeOH_QC_V1: Rt: 8.19 min, m/z 464.3 [M+H]+

m.p. (MeOH) 162-164 °C; ^{1}H NMR (600 MHz, CD_3OD) δ 7.77 (d, *J* 8.8 Hz, 2H), 7.51 (d, *J* 7.3 Hz, 1H), 7.47-7.44 (m, 2H), 7.01 (d, *J* 8.8 Hz, 2H), 6.93-6.89 (m, 2H), 6.64 (d, *J* 8.1 Hz, 1H), 4.51-4.47 (m, 1H), 4.39 (dd, *J* 11.4, 2.4 Hz, 1H), 4.18 (t, *J* 5.5 Hz, 2H), 4.08 (dd, *J* 11.6, 7.0 Hz, 1H), 3.92 (s, 3H), 3.73 (dd, *J* 14.1, 7.7 Hz, 1H), 3.65 (dd, *J* 14.1, 5.1 Hz, 1H), 2.80 (t, *J* 5.4 Hz, 2H), 2.36 (s, 6H), NH was not observed; ^{13}C NMR (150 MHz, CD_3OD) δ 170.2, 165.1, 163.2, 153.8, 145.1, 142.4, 140.1, 130.4, 130.3, 127.8, 124.3, 122.1, 119.3, 118.6, 115.5, 109.6, 73.2, 67.0, 67.0, 59.1, 53.9, 46.0, 41.1; *m/z* (ESI$^+$) 464 ([M+H]$^+$); HRMS (ESI$^+$) [$C_{26}H_{30}N_3O_5$] requires 464.21855, found 464.21871.

Supplementary Note 6

Synthesis of Abd-6:

(*R*)-(8-bromo-2,3-dihydrobenzo[b][1,4]dioxin-2-yl)methanamin (18)

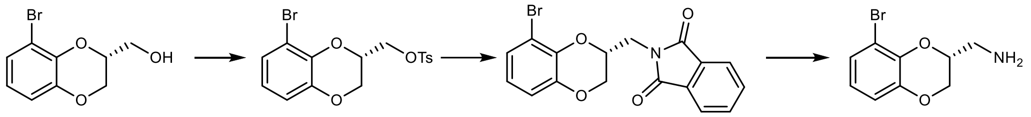

16 17 18

To a solution of ((*R*)-8-bromo-2,3-dihydro-benzo[1,4]dioxin-2-yl)-methanol (8.5 g, 34.7 mmol) in DCM (85 mL) was added Et_3N (18.9 mL, 139 mmol) dropwise over 10 min, TsCl (7.9 g, 41.6 mmol) was then added portion-wise over 10 min. The reaction mixture was stirred at room temperature for 3 h, then quenched with water and extracted with CH_2Cl_2 (2 x 100 mL). The combined organic layers were washed with water (2 x 200 mL) and brine (2 x 200 mL), dried (Na_2SO_4) and concentrated *in vacuo*. The crude product was partially-purified by column chromatography on silica gel (10% EtOAc:pet ether) to afford toluene-4-sulfonic acid (S)-(8-bromo-2,3-dihydrobenzo[b][1,4]dioxin-2-yl)methyl4-methylbenzenesulfonate**16** (10.7g) as a yellow solid, which was used directly in the subsequent reaction.

To a solution of toluene-4-sulfonic acid (*R*)-8-bromo-2,3-dihydro-benzo[1,4]dioxin-2-ylmethyl ester **16** (10.7 g) in DMF (90 mL) was added potassium phthalimide (7.44 g, 40.2 mmol), and the reaction stirred at 90 °C for 3 h. The mixture was cooled down, quenched with water and extracted with EtOAc (3 x 300 mL). The combined organic layers were washed with water (2 x 300 mL), brine (2 x 300 mL), dried (Na_2SO_4) and concentrated *in vacuo*. Purification on silica gel (15-20% EtOAc:pet ether) afforded 2-((*R*)-8-bromo-2,3-dihydro-benzo[1,4]dioxin-2-ylmethyl)-isoindole-1,3-dione **17** (8.70 g; 67% over 2 steps) as an off-white solid. $[\alpha]_D$ =−41.3 (c = 0.89, $CHCl_3$)

^1H NMR ($CDCl_3$, 400 MHz) 7.90-7.87 (m, 2H), 7.76-7.74 (m, 2H), 7.09 (dd, *J* 7.6, 1.6 Hz, 1H), 6.85-6.83 (m, 1H), 6.73 (t, *J* 8.2 Hz, 1H), 4.65-4.60 (m, 1H), 4.32 (dd, *J* 11.6, 2.4 Hz, 1H), 4.19-4.07 (m, 2H), 3.92 (dd, J 14.2, 5.4 Hz, 1H).

To a solution of 2-((*R*)-8-bromo-2,3-dihydro-benzo[1,4]dioxin-2-ylmethyl)-isoindole-1,3-dione **17** (8.7 g, 23.3 mmol) in EtOH (390 mL) was added hydrazine hydrate (11.6 g, 233 mmol). The reaction mixture was stirred at 90 °C under N_2 for 2 h. The reaction mixture was filtered through sintered funnel and the residue was washed with CH_2Cl_2 (2 x 200 mL). The combined filtrate and washings were concentrated *in vacuo* and the crude product (5 g) purified by reverse phase column chromatography (0.1% formic acid : MeCN) to afford (*R*)-(8-bromo-2,3-dihydrobenzo[b][1,4]dioxin-2-yl)methanamine **18** (3.5 g; 61%) as a yellow liquid.

AnalpH2_MeCN_UPLC_4min: Rt: 1.08 min, 244.1, 246.1 $[M+H]^+$.

^1H NMR ($CDCl_3$, 600 MHz) 7.10 (dd, *J* 7.9, 1.5 Hz, 1H), 6.83 (dd, *J* 8.3, 1.5 Hz, 1H), 6.71 (t, *J* 8.1 Hz, 1H), 4.29 (dd, *J* 11.3, 2.3 Hz, 1H), 4.24-4.20 (m, 1H), 4.04 (dd, *J* 11.4, 7.2 Hz, 1H), 3.05 (dd, *J* 13.4, 7.0 Hz, 1H), 3.01 (dd, *J* 13.6, 4.8 Hz, 1H), 1.43 (br s, 2H); ^{13}C NMR (150 MHz, $CDCl_3$) δ 144.2, 140.4, 125.2, 121.6, 116.3, 110.9, 65.9, 42.4.

(*R*)-N-((8-bromo-2,3-dihydrobenzo[b][1,4]dioxin-2-yl)methyl)tetrahydro-2H-pyran-4-carboxamide (19)

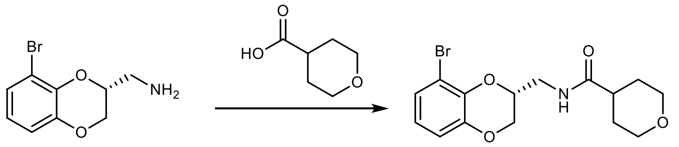

18 19

A solution of (*R*)-(8-bromo-2,3-dihydrobenzo[b][1,4]dioxin-2-yl)methanamin **18** (120 mg, 0.494 mmol) in DMF (3 mL) was treated sequentially with *N*,*N*-diisopropylethylamine (345

µL, 1.98 mmol), tetrahydro-pyran-4-carboxylic acid (77 mg, 0.593 mmol), and HATU (263 mg, 0.692 mmol), and the reaction was stirred at room temperature for 16 h. The reaction was diluted with EtOAc (25 mL) and washed with a 50/50 solution of water and brine (3 x 50 mL). The organic phase was dried (Na_2SO_4) and concentrated *in vacuo.* Purification by column chromatography on silica gel (5% MeOH:CH_2Cl_2) afforded (*R*)-*N*-((8-bromo-2,3-dihydrobenzo[b][1,4]dioxin-2-yl)methyl)tetrahydro-2H-pyran-4-carboxamide **19** (156 mg, 89%) as a white solid. $[\alpha]_D$ =+34.3 (c = 0.92, $CHCl_3$); m.p. (MeOH) 154-155 °C; ^{1}H NMR (600 MHz, $CDCl_3$) δ 7.12 (dd *J* 7.9, 1.3 Hz, 1H), 6.85 (dd, *J* 8.1, 1.3 Hz, 1H), 6.75 (t, *J* 8.1 Hz, 1H), 5.98 (br s, 1H), 4.36-4.35 (m, 1H), 4.31 (dd, *J* 11.6, 2.3 Hz, 1H), 4.04-4.02 (m, 2H), 3.65 (dd, *J* 11.6, 7.2 Hz, 1H), 3.81 (ddd, *J* 14.4, 6.7, 3.7 Hz, 1H), 3.49-3.41 (m, 3H), 2.43-2.38 (m, 1H), 1.86-1.78 (m, 4H); ^{13}C NMR (150 MHz, $CDCl_3$) δ 174.6, 144.0, 139.8, 125.3, 122.1, 116.5, 110.8, 72.6, 67.2, 67.2, 65.6, 42.1, 39.3, 29.2, 29.2; *m/z* (ESI$^+$) 356 ([M+H]$^+$); HRMS (ESI$^+$) [$C_{15}H_{19}BrNO_4$] requires 356.04930, found 356.04920.

(*R*)-N-((8-(5-amino-6-methoxypyridin-2-yl)-2,3-dihydrobenzo[b][1,4]dioxin-2-yl)methyl)tetrahydro-2H-pyran-4-carboxamide (21)

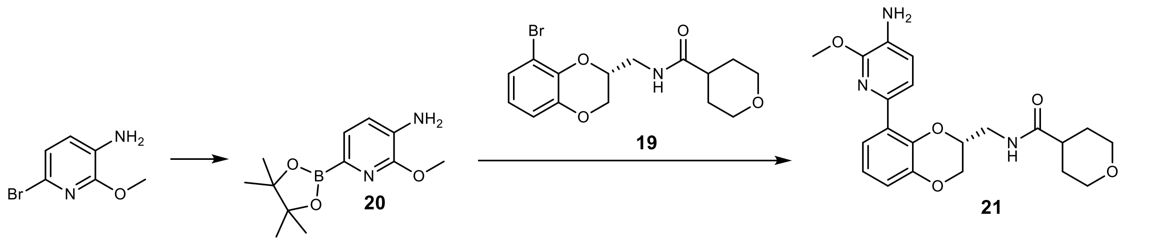

3-Amino-6-bromo-2-methoxypyridine (200 mg, 0.983 mmol), bis(pinacolato)diboron (375 mg, 1.47 mmol), KOAc (290 mg, 2.95 mmol) and Pd(dppf)Cl_2 (72 mg, 0.098 mmol) were added sequentially to a vial and degassed with N_2 for 5 min. Degassed 1,4-dioxane (10 mL) was then added, the vial sealed and heated to 100 °C for 18 h. The mixture was cooled down and passed through celite, using EtOAc as an eluent. The filtrate was concentrated *in vacuo* and the 2-methoxy-6-(4,4,5,5-tetramethyl-1,3,2-dioxaborolan-2-yl)pyridin-3-amine **20** was obtained quantitatively. $[\alpha]_D$ =+21.9 (c = 0.78, $CHCl_3$). ^{1}H NMR (400 MHz, MeOD) δ 7.25 (d, *J* 7.6 Hz, 1H), 6.85 (d, *J* 7.6 Hz, 1H), 4.02 (s, 3H), 1.20 (s, 12H), NHs were not observed.(*R*)-N-*((*8-bromo-2,3-dihydrobenzo[b][1,4]dioxin-2-yl)methyl)tetrahydro-2H-pyran-4-carboxamide **19** (80 mg, 0.225 mmol), 2-methoxy-6-(4,4,5,5-tetramethyl-1,3,2-dioxaborolan-2-yl)pyridin-3-amine **20** (111 mg, 0.450 mmol), K_2CO_3 (93 mg, 0.675 mmol) and Pd(dppf)Cl_2 (8 mg, 0.011 mmol) were added sequentially to a vial and degassed with N_2 for 5 min. Degassed 1,4-dioxane:water (10:1, 10 mL) was then added, the vial sealed and heated to 100 °C for 18 h. The mixture was cooled down, diluted with EtOAc (30 mL), and washed with a 50/50 solution of water and brine (30 mL). The organic phase was dried (Na_2SO_4) and concentrated *in vacuo.* Purification by column chromatography on silica gel (8% MeOH:CH_2Cl_2) afforded (*R*)-*N*-((8-(5-amino-6-methoxypyridin-2-yl)-2,3-dihydrobenzo[b][1,4]dioxin-2-yl)methyl)tetrahydro-2H-pyran-4-carboxamide **21** as an orange glassy solid (83 mg, 92%); m.p. (MeOH) 171-173 °C; ^{1}H NMR (600 MHz, $CDCl_3$) δ 7.50 (dd, *J* 7.7, 1.5 Hz, 1H), 7.33 (d, *J* 7.7 Hz, 1H), 6.94 (t, *J* 8.1 Hz, 1H), 6.91 (d, *J* 7.9 Hz, 1H), 6.86 (dd, *J* 8.1, 1.7 Hz, 1H), 5.81 (br s, 1H), 4.39-4.35 (m, 1H), 4.33 (dd, J 11.4, 2.4 Hz, 1H), 4.06 (s, 3H), 4.02 (dd, J 11.3, 6.7, 1H), 3.99-3.97 (m, 2H), 3.89 (br s, 2H), 3.78 (ddd, J 14.3, 6.8, 4.0 Hz, 1H), 3.44 (ddd, J 14.1, 7.7, 5.1 Hz, 1H), 3.38 (tt, J 11.4, 3.0 Hz, 2H), 2.30-2.25 (m, 1H), 1.70-1.66 (m, 4H); ^{13}C NMR (150 MHz, $CDCl_3$) δ 174.5, 152.1, 143.3, 140.3, 139.9, 129.7, 129.2, 122.7, 121.3, 119.8, 118.2, 116.4, 75.0, 67.2, 65.3, 42.1, 39.5, 29.2, 24.9; *m/z* (ESI$^+$) 400 ([M+H]$^+$); HRMS (ESI$^+$) [$C_{21}H_{26}N_3O_5$] requires 400.18725, found 400.18740.

(*R*)-*N*-((8-(5-((3-((dimethylamino)methyl)phenyl)amino)-6-methoxypyridin-2-yl)-2,3-dihydrobenzo[b][1,4]dioxin-2-yl)methyl)tetrahydro-2H-pyran-4-carboxamide (Abd-6)

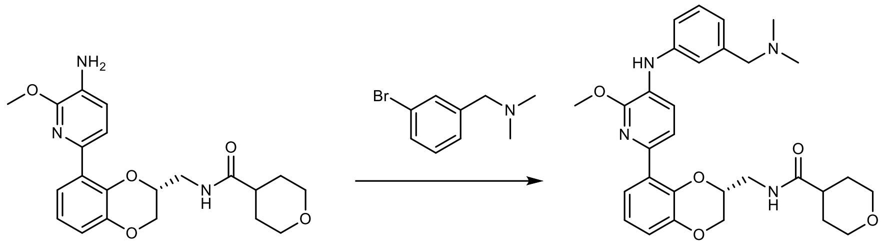

21 **Abd-6**

(*R*)-*N*-((8-(5-amino-6-methoxypyridin-2-yl)-2,3-dihydrobenzo[b][1,4]dioxin-2-yl)methyl)tetrahydro-2H-pyran-4-carboxamide **21** (65 mg, 0.163 mmol), Cs_2CO_3 (160 mg, 0.489 mmol), 1-(3-bromophenyl)-*N*,*N*-dimethylmethanamine (38 mg, 0.179 mmol), XPhos (8 mg, 0.016 mmol), $Pd(OAc)_2$ (2 mg, 0.008 mmol), were added sequentially to a vial and degassed with N_2 for 5 min. Degassed 1,4-dioxane (4 mL) was then added, the vial sealed and heated to 100 °C for 18 h. The mixture was cooled down, diluted with EtOAc (30 mL), and washed with a 50/50 solution of water and brine (30 mL). The organic phase was dried (Na_2SO_4) and concentrated *in vacuo.* Purification by column chromatography on silica gel (20% MeOH:CH_2Cl_2) afforded (*R*)-*N*-((8-(5-((3-((dimethylamino)methyl)phenyl)amino)-4-methoxypyridin-2-yl)-2,3-dihydrobenzo[b][1,4]dioxin-2-yl)methyl)tetrahydro-2H-pyran-4-carboxamide **Abd-6** as a pale yellow solid (78 mg, 90%). $[\alpha]_D$ =+17.2 (c = 0.81, $CHCl_3$); m.p. (MeOH) 188-189°C; ^1H NMR (600 MHz, CD_3OD) δ 7.54-7.52 (m, 3H), 7.27 (dd, *J* 8.8, 7.5 Hz, 1H), 7.14-7.13 (m, 2H), 6.92 (ddd, *J* 7.5, 1.3, 1.1 Hz, 1H), 6.88 (t, *J* 7.9 Hz, 1H), 6.82 (dd, *J* 7.9, 1.7 Hz, 1H), 4.56 (br s, 1H), 4.37-4.34 (1H, m, 1H), 4.32 (dd, *J* 11.2, 2.4 Hz, 1H), 4.07 (s, 3H), 4.01 (dd, *J* 11.4, 6.8 Hz, 1H), 3.89-3.86 (m, 2H), 3.57 (dd, *J* 14.3, 7.4 Hz, 1H), 3.49-3.46 (m, 3H), 3.34 (ddd, *J* = 11.7, 2.4, 2.0 Hz, 1H), 2.43-2.37 (m, 1H), 2.28 (s, 6H), 1.71-1.64 (m, 2H), 1.59-1.56 (m, 2H), NHs were not observed; ^{13}C NMR (150 MHz, CD_3OD) δ 178.0, 154.4, 145.0, 144.0, 142.6, 142.1, 130.4, 130.3, 128.4, 123.9, 123.7, 121.9, 121.2, 121.0, 119.6, 119.2, 117.4, 73.1, 68.4, 68.4, 66.8, 65.2, 54.0, 49.7, 45.4, 43.2, 40.9, 30.5, 30.5; *m/z* (ESI^+) 533 ($[M+H]^+$); HRMS (ESI^+) [$C_{30}H_{37}N_4O_5$] requires 533.27640, found 533.27621.

Supplementary Note 7

Synthesis of Abd-7:

3-chloro-6-(2,3-dihydrobenzo[b][1,4]dioxin-5-yl)-2-methoxypyridine (23)

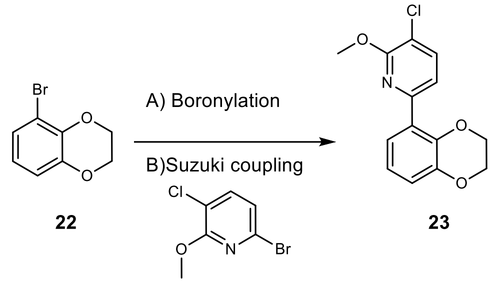

A solution of 5-bromo-2,3-dihydro-benzo[1,4]dioxine **22** (298 mg, 1.40 mmol) in 1,4-dioxane (10 mL) was purged with N_2 for 10 min. KOAc (342 mg, 3.49mmol), bis(pinacolato)diboron (531 mg, 2.09 mmol) and Pd(dppf)Cl_2 (114 mg, 0.140 mmol) were added and the mixture purged again with N_2 for 10 min, and heated at 110 °C for 2.5 h. The reaction was then cooled before sequential addition of 6-bromo-3-chloro-2-methoxy-pyridine (341 mg, 1.53 mmol), $Pd(PPh_3)_4$ (161 mg, 0.140 mmol), K_2CO_3 (386 mg, 2.79 mmol) and water (1 mL). The solution was purged with N_2 for 5 min and then heated at 110 °C for 2 h. The mixture was cooled down and concentrated *in vacuo*. The residue was purified by column chromatography on silica gel, and then by prep-HPLC to afford 3-chloro-6-(2,3-dihydrobenzo[b][1,4]dioxin-5-yl)-2-methoxypyridine **23** (125 mg, 32%) as a white solid.
AnalpH2_MeOH_4min_V1: Rt: 3.46 min, m/z 278.2/280.2 $[M+H]^+$.
m.p. (MeOH) 109-110 °C; ^{1}H NMR (600 MHz, $CDCl_3$) δ 7.64 (d, *J* 7.9 Hz, 1H), 7.53 (dd, *J* 7.0, 2.2 Hz, 1H), 7.51 (d, *J* 7.9 Hz, 1H), 6.97-6.93 (m, 2H), 4.34-4.32 (m, 4H), 4.10 (s, 3H); ^{13}C NMR (150 MHz, $CDCl_3$) δ 158.4, 150.1, 143.9, 141.7, 138.0, 127.8, 122.9, 121.0, 118.5, 117.9, 116.2, 64.4, 64.0, 54.0. *m/z* (ESI^+) 278 ($[M+H]^+$); HRMS (ESI^+) [$C_{14}H_{14}NCl$] requires 278.05785, found 278.05750.

6-(2,3-dihydrobenzo[b][1,4]dioxin-5-yl)-N-(3-((dimethylamino)methyl)phenyl)-2-methoxypyridin-3-amine (Abd-7)

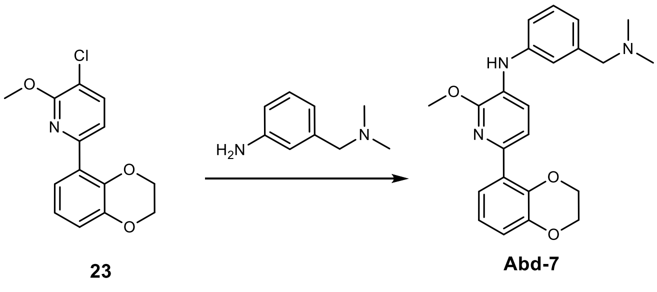

To a solution of 3-chloro-6-(2,3-dihydro-benzo[1,4]dioxin-5-yl)-2-methoxy-pyridine **23** (111 mg, 0.400 mmol) in 1,4-dioxane (5 mL) was added 4-dimethylaminomethyl-phenylamine (60 mg, 0.400 mmol), $Pd(OAc)_2$ (9 mg, 0.040 mmol), XPhos (57 mg, 0.120 mmol) and NaO^{t}Bu (57 mg, 0.600 mmol) . The reaction mixture was degassed with N_2 for 10 min, heated at 100 °C for 21 h, cooled to room temperature, partitioned between water and EtOAc and the organic phase separated. The aqueous phase was extracted with EtOAc and the combined organic phases dried (Na_2SO_4) and concentrated *in vacuo*. Purification by prep HPLC column chromatography yielded 6-(2,3-dihydrobenzo[b][1,4]dioxin-5-yl)-*N*-(3-((dimethylamino)-methyl)phenyl)-2-methoxypyridin-3-amine **Abd-7** (46 mg, 29%) as a white solid. AnalpH2_MeOH_QC_V1: Rt: 6.17 min, m/z 392.3 $[M+H]^+$; AnalpH9_MeOH_QC_V1: Rt: 8.62 min, m/z 392.4 $[M+H]^+$.

m.p. (MeOH) 115-117 °C; ^{1}H NMR (600 MHz, CD_3OD) δ 7.50 (dd, *J* 7.7, 1.7 Hz, 1H), 7.48 (s, 2H), 7.22 (d, *J* 8.4 Hz, 2H), 7.14 (d, *J* 8.6 Hz, 2H), 6.84 (t, *J* 7.9 Hz, 1H), 6.79 (dd, *J* 7.9, 1.7 Hz, 1H), 4.31-4.27 (m, 4H), 4.06 (s, 3H), 3.43 (s, 2H), 2.25 (s, 6H), NH was not observed; ^{13}C NMR (150 MHz, CD_3OD) δ 154.4, 145.5, 143.4, 143.0, 142.9, 131.9, 131.3, 130.3, 128.2, 123.5, 121.7, 121.2, 119.6, 119.3, 117.6, 65.7, 65.4, 64.5, 53.9, 45.2; *m/z* (ESI^+) 392 ($[M+H]^+$); HRMS (ESI^+) [$C_{25}H_{26}N_3O_3$] requires 392.19742, found 392.19721.

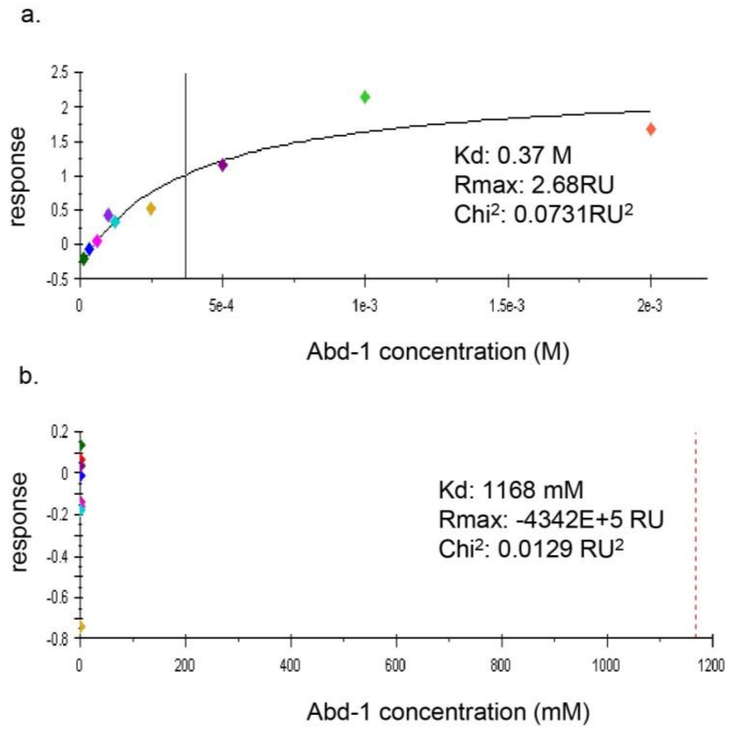

Supplementary Figure 1: Kd for Abd-1 and 2 binding HRAS^{G12V}-GTPγS using SPR

The binding of **Abd-1** was analyzed by SPR with HRAS^{G12V}-GTPYS (**panel a**) and predicted with HRAS-GDP (**panel b**). In the second panel the dose response experiment did not generate a curve. The Kd value is only a prediction generated by the Biacore SPR T-100 software. The data are from a single experimental analysis.

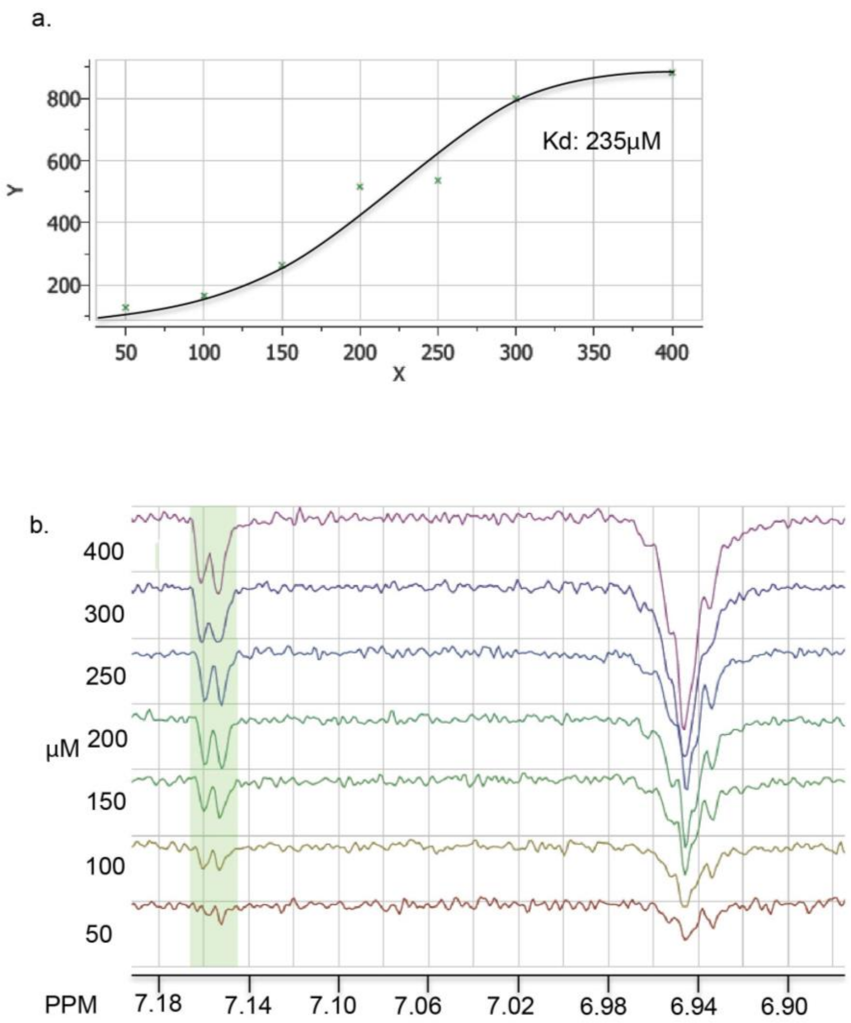

Supplementary Figure 2: Kd for Abd-2 with HRAS^{G12V}-GTPγS using NMR waterLOGSY

The Kd for Abd-2 was generated using NMR waterLOGSY binding curve (**panel a**) showing NMR signal (y axis) against mM concentration (x axis). **Panel b** shows the waterLOGSY NMR spectra from the Abd-2 titration ranging from 50 to 400µM compound. The dissociation constant of Abd-2 binding to HRAS^{G12V}-GTPγS was determined from the curve generated by plotting the reduction of the NMR signal from a selected peak (depicted in green) during the compound titration against the protein. The data are from a single experimental analysis.

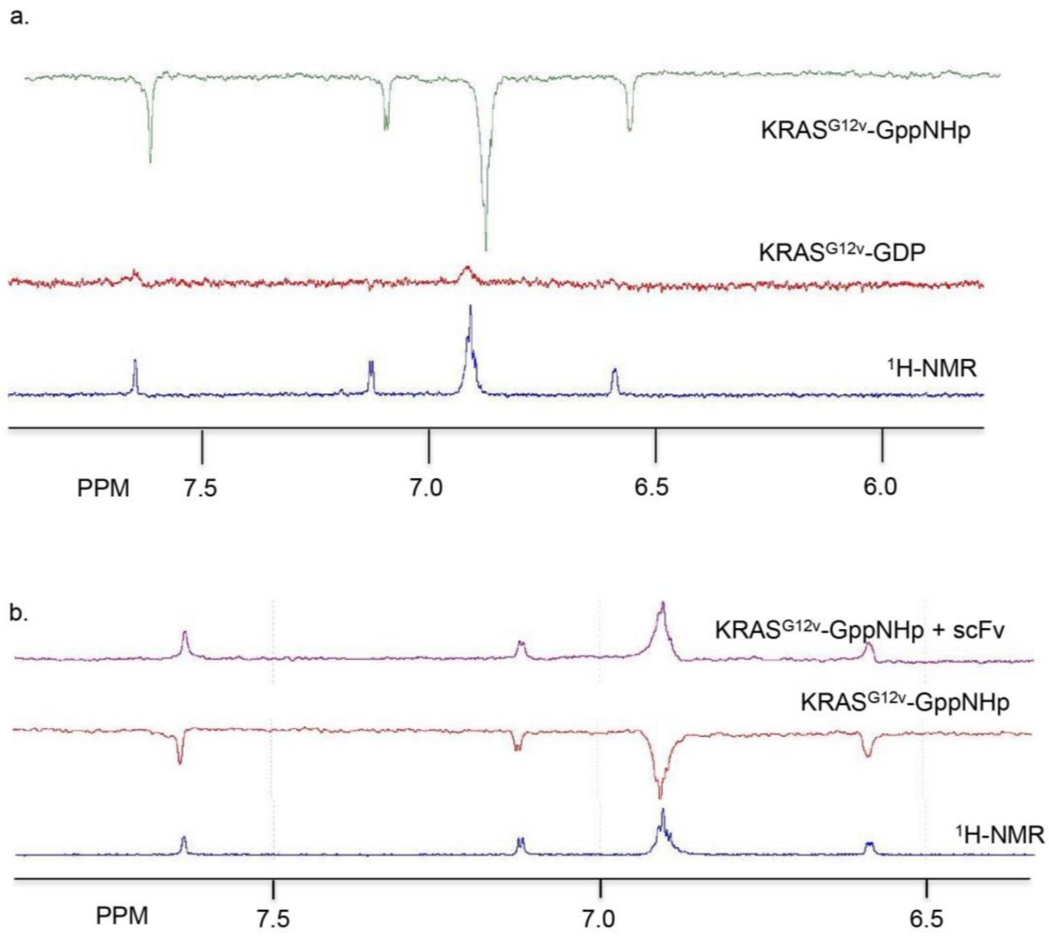

Supplementary Figure 3: Abd-2 WaterLOGSY NMR with and without anti RAS-scFV

Abd-2 was analyzed by waterLOGSY. **Panel a**, WaterLOGSY NMR shows preferential binding of Abd-2 to KRAS^{G12V}-GppNHp (upper trace, in green) but poor binding to KRAS-GDP (middle trace, in red). The proton NMR spectrum of Abd-2 in the absence of protein is shown in the lower trace. **Panel b**, WaterLOGSY of Abd-2 with KRAS^{G12V}-GppNHp in the presence or absence of scFv competitor respectively top (no binding of Abd-2 to the KRAS^{G12V}-GppNHp + scFv complex) and middle spectra (binding to KRAS^{G12V}-GppNHp without scFv) in purple and red respectively. The lower spectra is the proton NMR of Abd-2 in the absence of protein. Results from **panel b** confirm the interference of binding by the anti-RAS scFv.

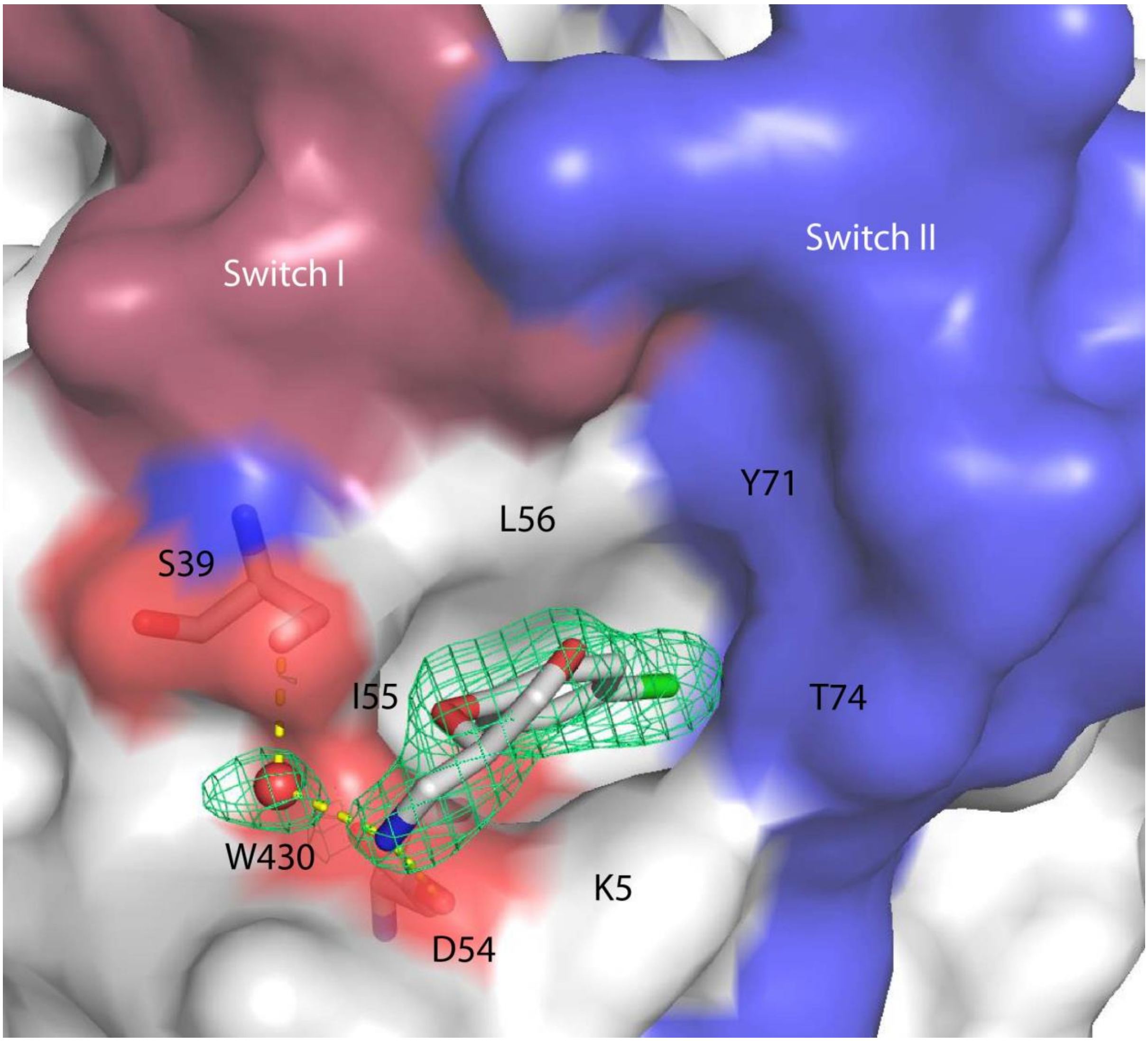

Supplementary Figure 4: Abd-3 binding in the hydrophobic pocket of KRAS

The region around the binding pocket of $KRAS^{Q61H}$ showing binding mode of Abd-3 and the amino-acids surrounding the pocket. The switch regions are highlighted in purple (switch I) and blue (switch II).

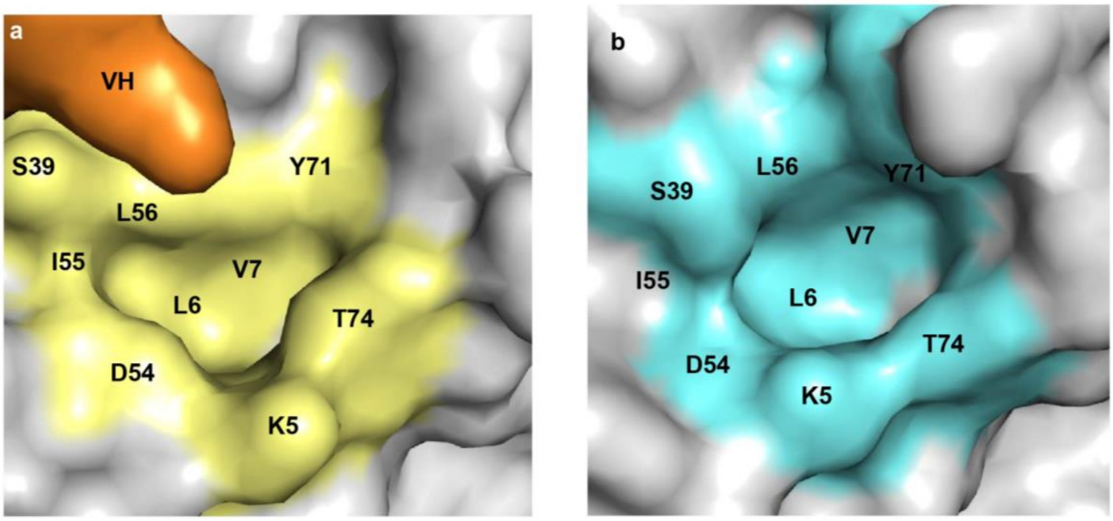

Supplementary Figure 5: $HRAS_{166}^{G12V}$-Fv and $HRAS_{166}^{G12V}$ surface comparison

The structures of $HRAS_{166}^{G12V}$-GppNHp-Fv and $HRAS_{166}^{G12V}$-GppNHp are from PDB 2VH5 and 4EFM respectively. **Panel a**, $HRAS_{166}^{G12V}$-Fv protein surface from 2VH5 around the identified pocket with amino acid residues identified in yellow. **Panel b**, $HRAS^{G12V}$-GppNHp protein surface from 4EFM around the identified pocket with amino acid residues identified in cyan.

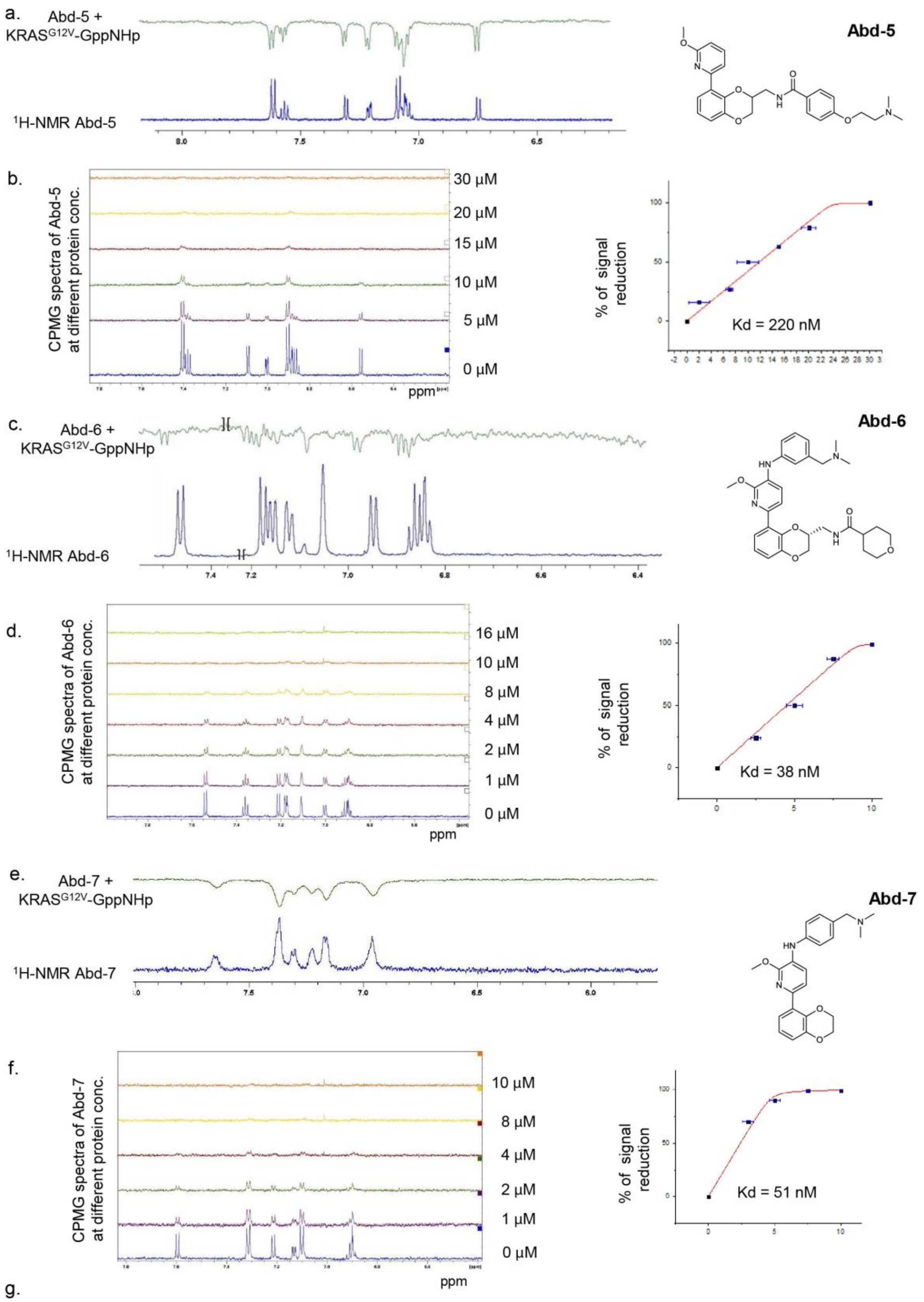

g.

	Abd-4	Abd-5	Abd-6	Abd-7
**KRAS Kd (uM)**	-----	0.220	0.038	0.051
**M.W**	356.4	478.5	532.6	391.5
**L.E.**	-----	0.27	0.26	0.34
**Solubility (uM)**	150	280	47	36

Supplementary Figure 6: Abd-7 Binding in WaterLOGSy and Kd calculations in CPMG
WaterLOGSY and CPMG NMR orthogonal analysis of Abd-5 (**panel a, b**), Abd-6 (**c, d**), and Abd-7 (**e, f**) with GST-KRAS^{G12V}-GppNHp. For each waterLOGSY profile, the proton NMR spectrum of the compound is shown together the spectra of the compound after incubation with the KRAS (the NOE transfers occurs when the compounds displace those waters upon binding to the protein). The Kd of interaction of the compounds come from fitting a binding curve (right of **panels b, d, f**) of a CPMG titration (shown on the left of panels **b, d, f**). The calculated Kd is shown for each compound. **Panel g** shows a table of molecular weight (MW), Kd, Solubility and ligand efficiency (LE). Each experiment was repeated at least three times. Where error bars are presented, they correspond to mean values ± SD of experimental repeats (**b, d and f**).

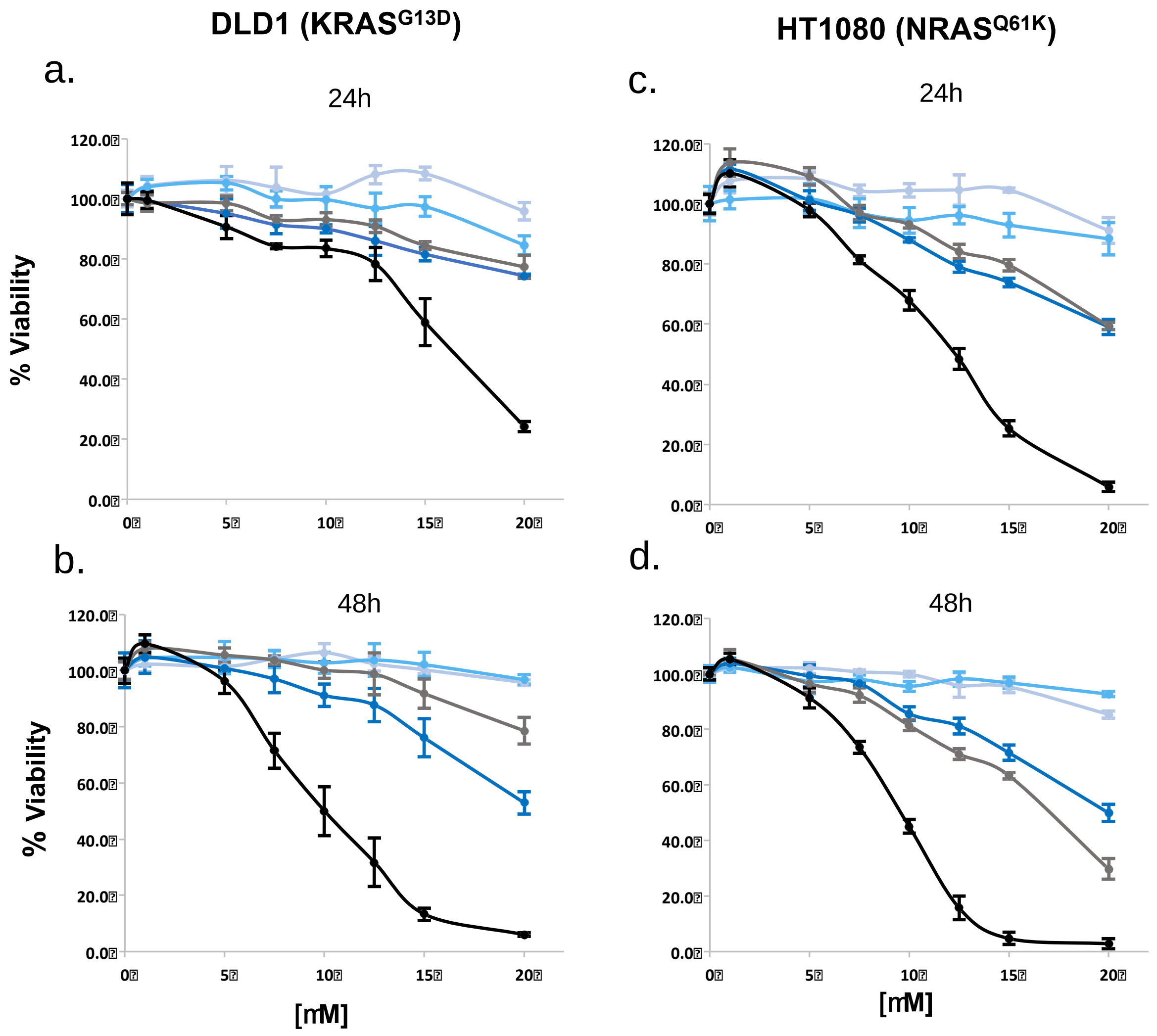

e.

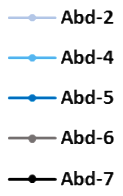

f.

	DLD-1 IC50		HT1080 IC50	
	48h	72h	48h	72h
**Abd-7**	9.8 mM	8.0 mM	9.4 mM	10.0 mM

Supplementary Figure 7: The effect of Abd compounds on the viability of cancer cells

The effect of the chemical series on the viability of human cancer cells lines DLD-1 (a colorectal cancer cell line with mutant KRAS^{G13D}) and HT1080 (a fibrosarcoma cell line with mutant NRAS^{Q61K}). The cells were treated with a dose range from 0mM to 20mM of either Abd-2, Abd-4, Abd-5, Abd-6 and Abd-7 and incubated for 24 or 48 hours. Cell viability was assessed using CellTitreGlo. In each case, the data are normalized to cells treated with DMSO only. **Panels a** and **b** are DLD-1 viability and **panels c** and **d** are HT1080 viability. Panel **e** shows the colour coding for the different compounds. The IC$_{50}$ for Abd-7 at 48 & 72 hours are shown in **panel f**. Each experiment was repeated at least four times (a to d). Where error bars are presented, they correspond to mean values ± SD of biological repeats.

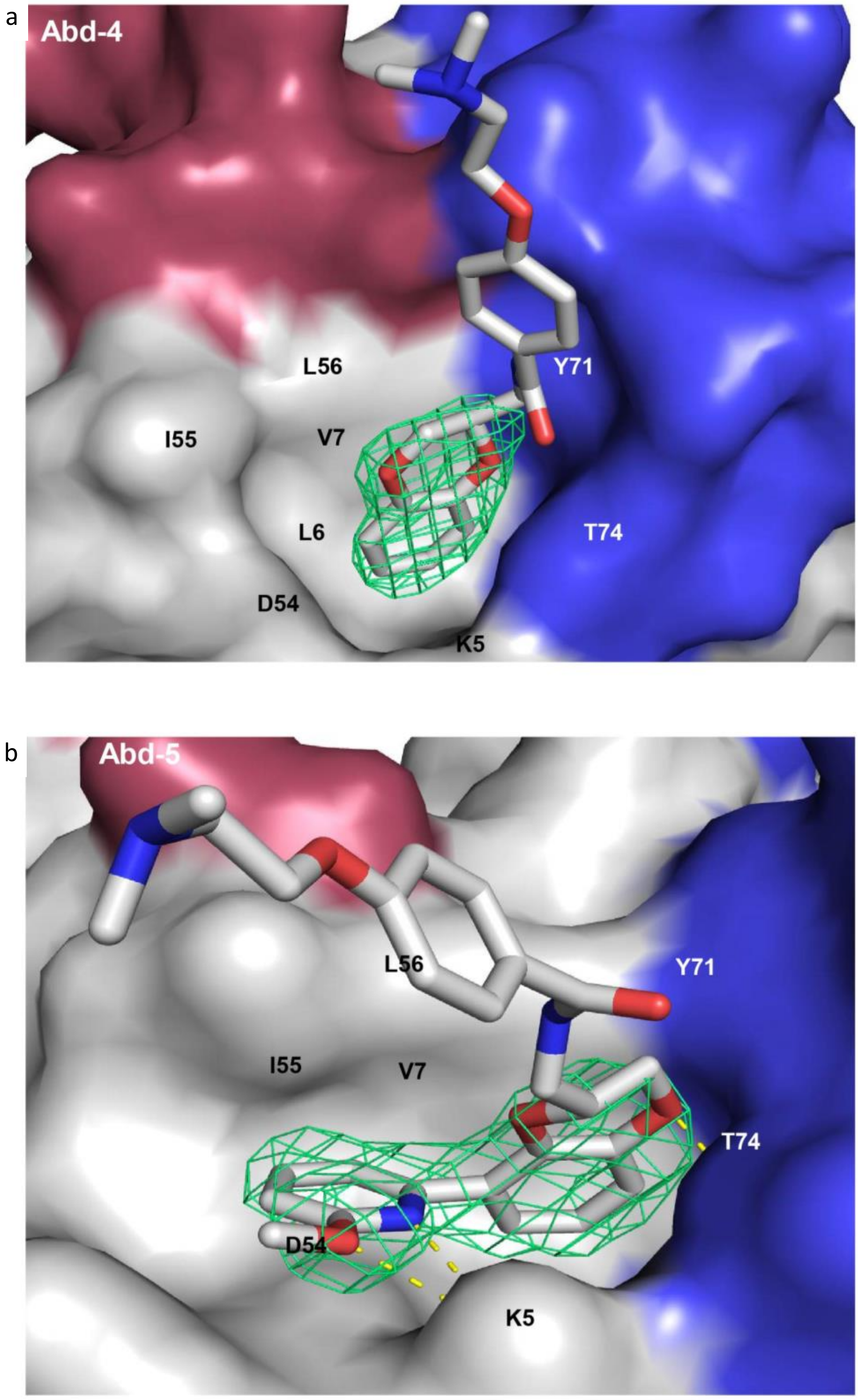

a
Abd-4
L56
Y71
I55
V7
L6
T74
D54
K5
b
Abd-5
L56
Y71
I55
V7
T74
D54
K5

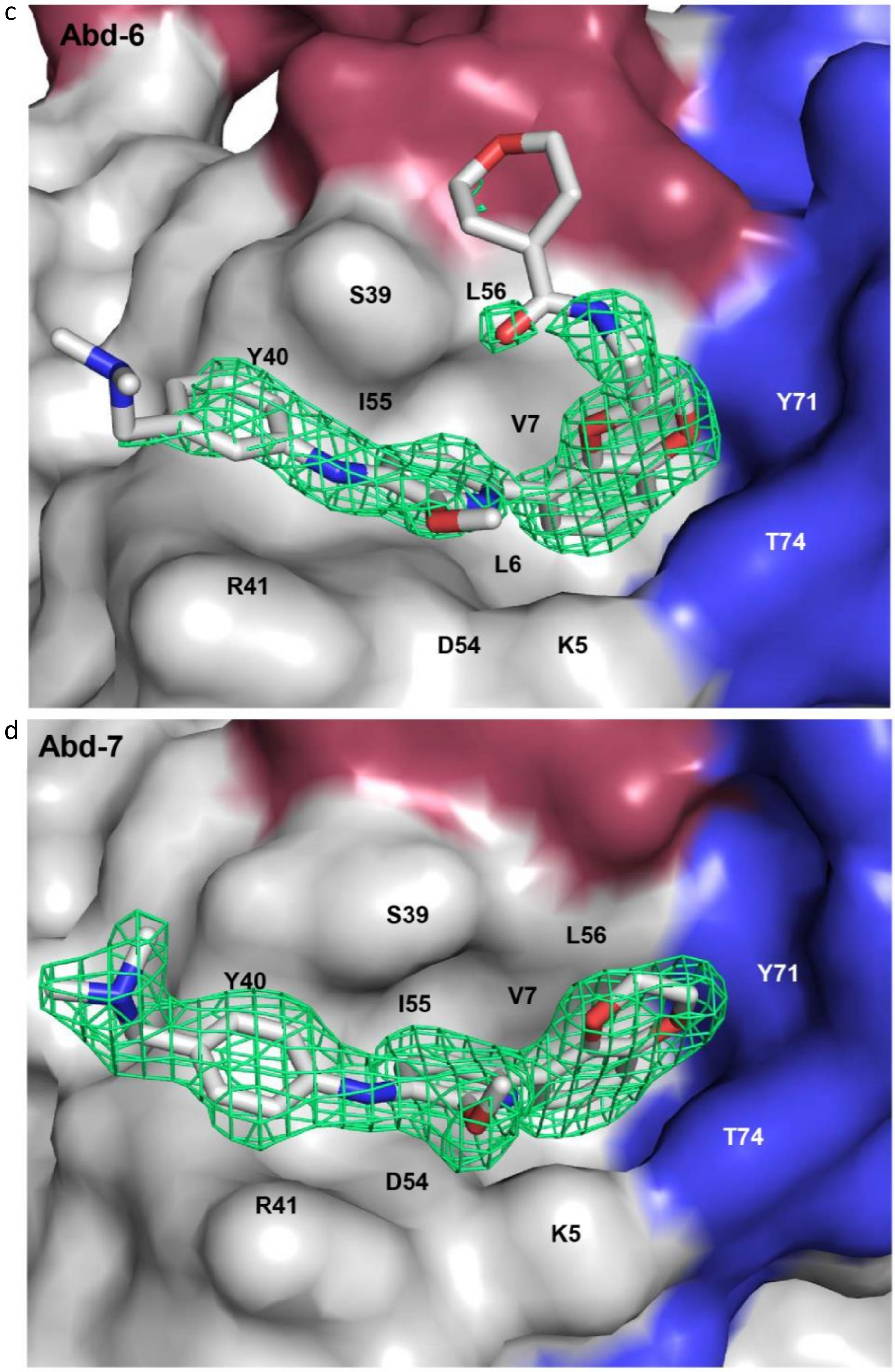

Supplementary Figure 8: Expanded views of crystal data with Abd-4, 5, 6 and 7.

Panel a, KRAS accommodating the benzodioxane ring in Abd-4 (K5, L6, V7, D54, I55, L56, Y71 and T74). **Panel b**, KRAS accommodating three rings in Abd-5 by rotation of D54 (K5, L6, V7, D54, I55, L56, Y71 and T74). **Panels c** and **d.** KRAS accommodating a further aniline type functionality in Abd-6 and Abd-7 (K5, L6, V7, S39, Y40, R41, D54, I55, L56, Y71 and T74.

a

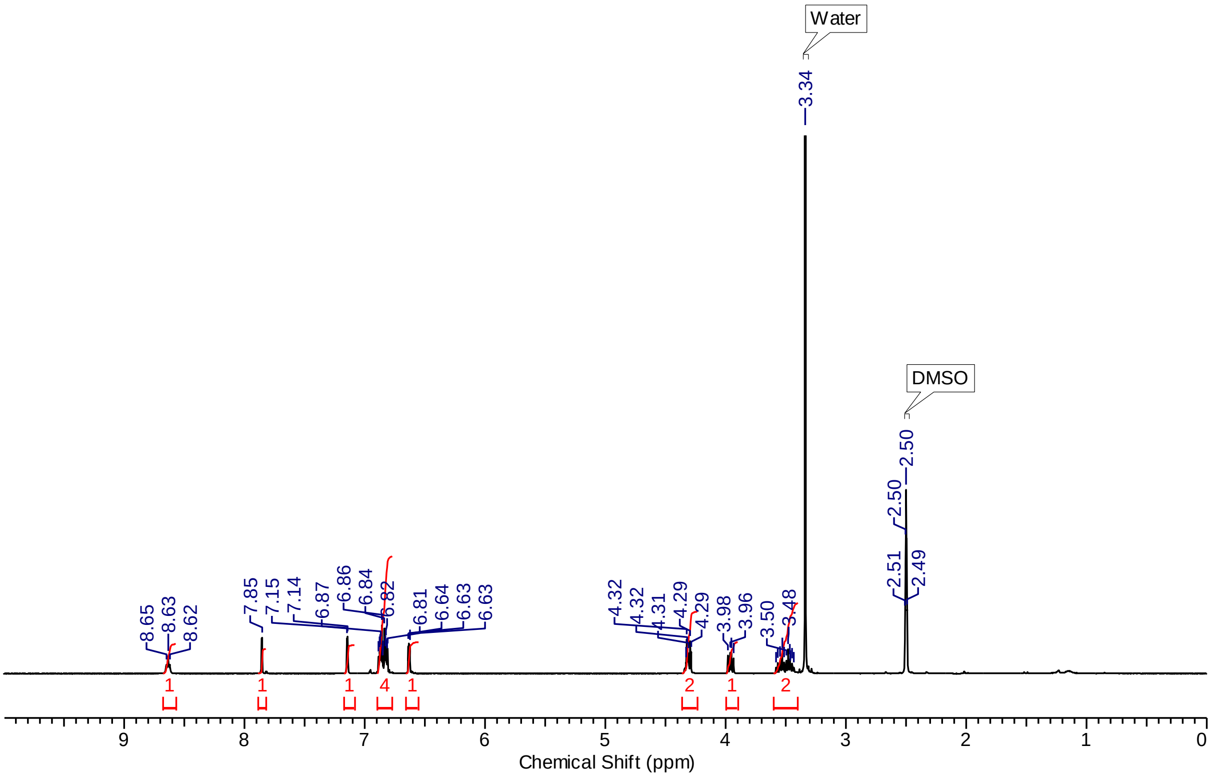

Water
3.34
DMSO
2.50
2.50
2.51
2.49
8.65
8.63
8.62
7.85
7.15
7.14
6.87
6.86
6.84
6.82
6.81
6.64
6.63
6.63
4.32
4.32
4.31
4.29
4.29
3.98
3.96
3.50
3.48
1
1
1
4
1
2
1
2
9
8
7
6
5
4
3
2
1
0
Chemical Shift (ppm)

b

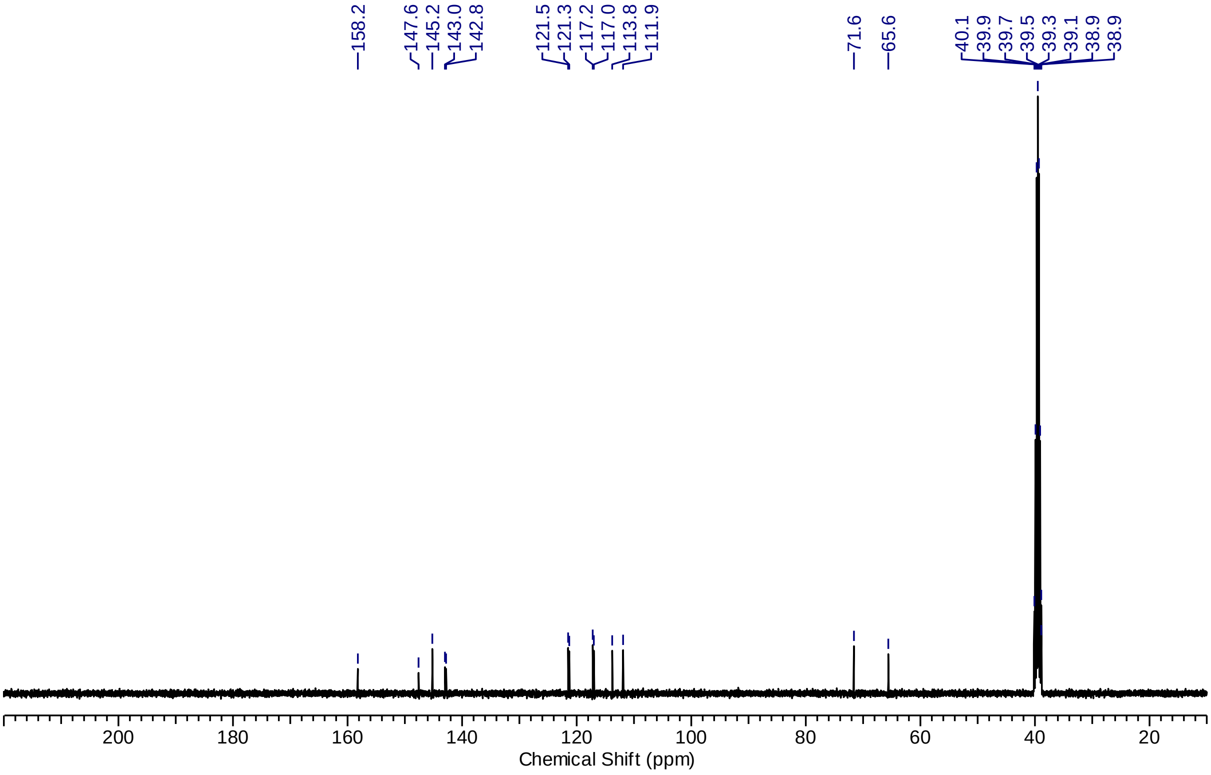

158.2
147.6
145.2
143.0
142.8
121.5
121.3
117.2
117.0
113.8
111.9
71.6
65.6
40.1
39.9
39.7
39.5
39.3
39.1
38.9
38.9
200
180
160
140
120
100
80
60
40
20
Chemical Shift (ppm)

c

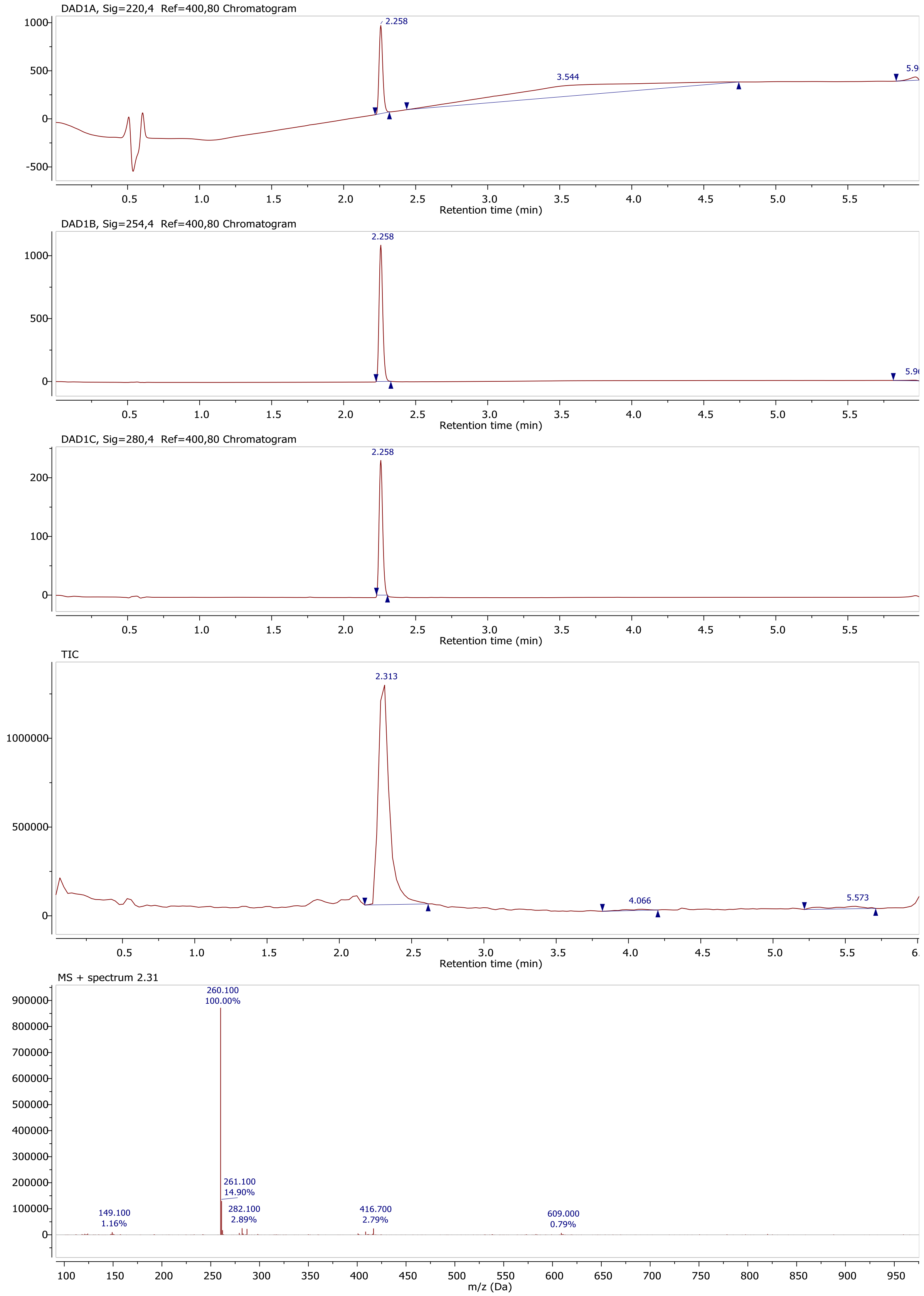

Supplementary Figure 9: ^{1}H NMR, ^{13}C NMR and LCMS of Abd-2.

Data were recorded on Bruker Avance spectrometers (AVII400 or AVIII400) in the deuterated solvent stated. The field was locked by external referencing to the relevant

deuteron resonance. Chemical shifts (δ) are reported in parts per million (ppm) referenced to the solvent peak. **Panel a**: ^{1}H NMR spectra. **Panel b**: ^{13}C NMR spectra. **Panel c**: LCMS.

a

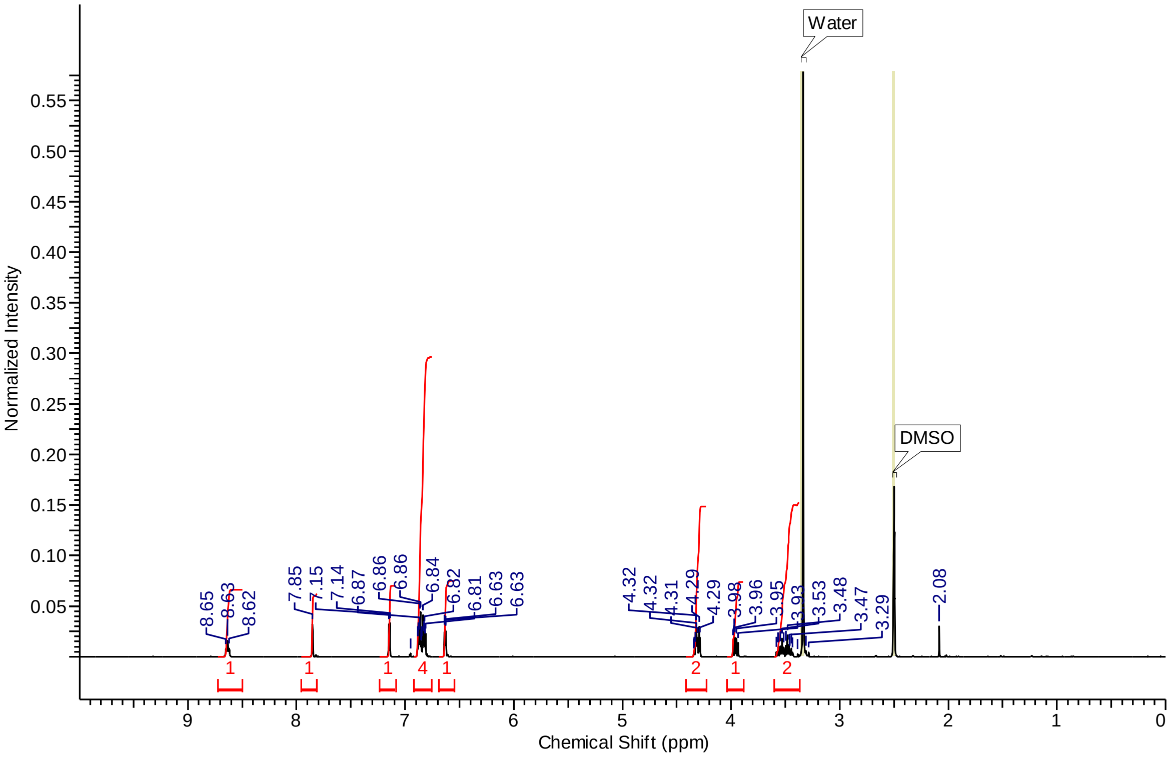

Water
DMSO
Normalized Intensity
Chemical Shift (ppm)

b

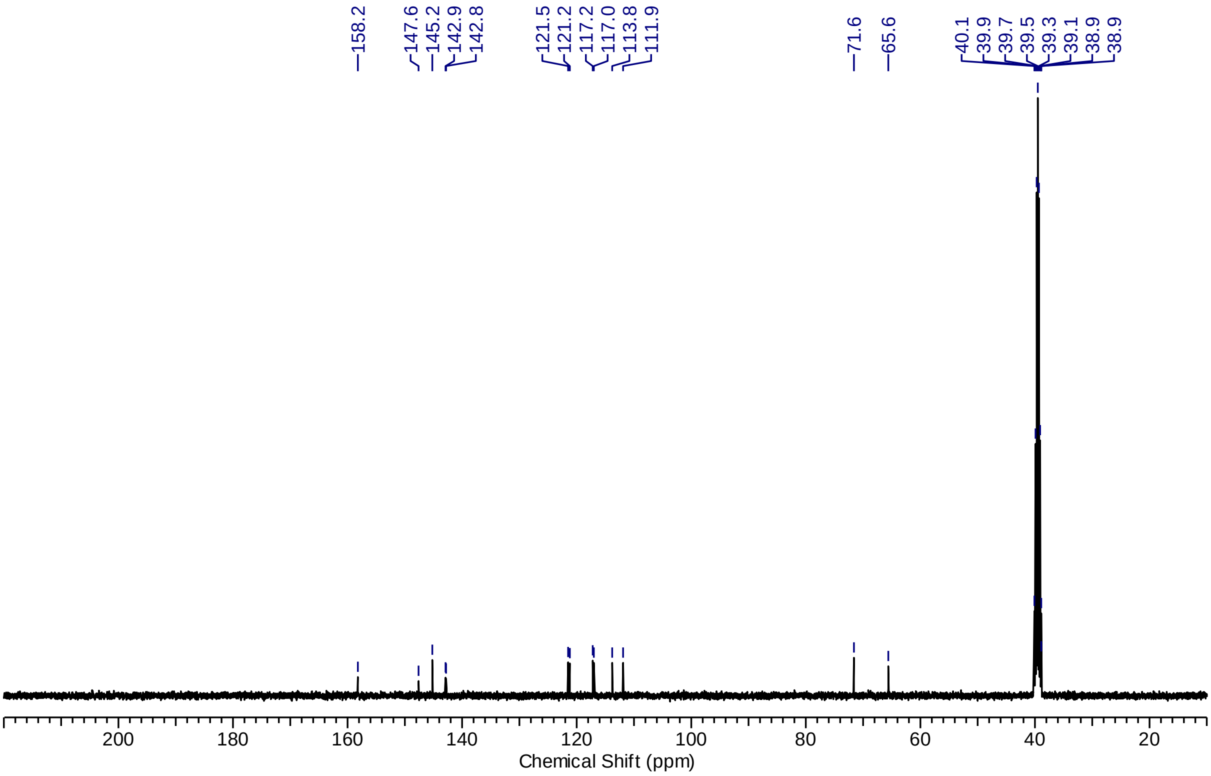

158.2
147.6
145.2
142.9
142.8
121.5
121.2
117.2
117.0
113.8
111.9
71.6
65.6
40.1
39.9
39.7
39.5
39.3
39.1
38.9
38.9
Chemical Shift (ppm)

c

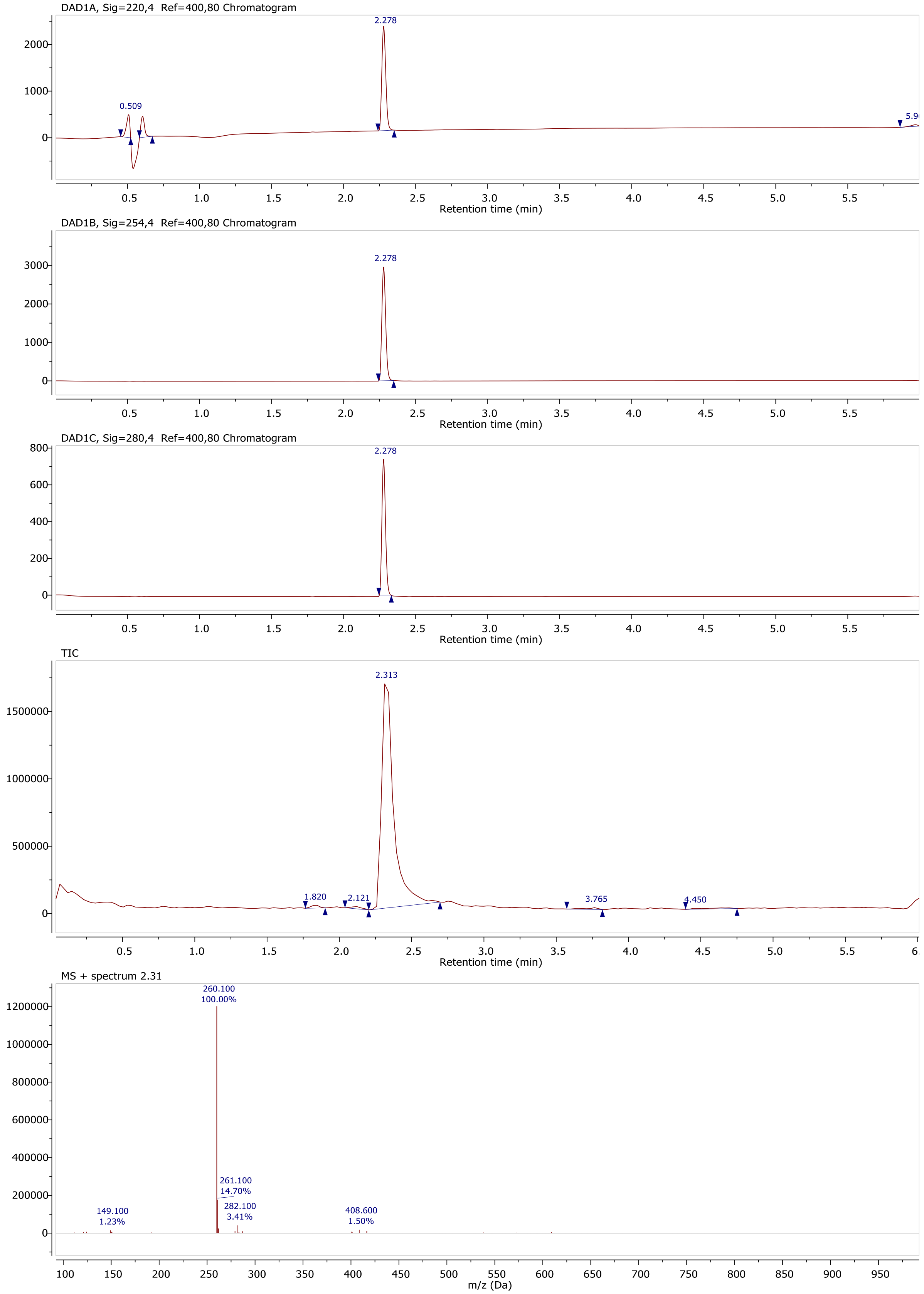

Supplementary Figure 10: ^{1}H NMR, ^{13}C NMR and LCMS of Abd-2a.

Data were recorded on Bruker Avance spectrometers (AVII400 or AVIII400) in the deuterated solvent stated. The field was locked by external referencing to the relevant

deuteron resonance. Chemical shifts (δ) are reported in parts per million (ppm) referenced to the solvent peak. **Panel a**: ^{1}H NMR spectra. **Panel b**: ^{13}C NMR spectra. **Panel c**: LCMS.

a

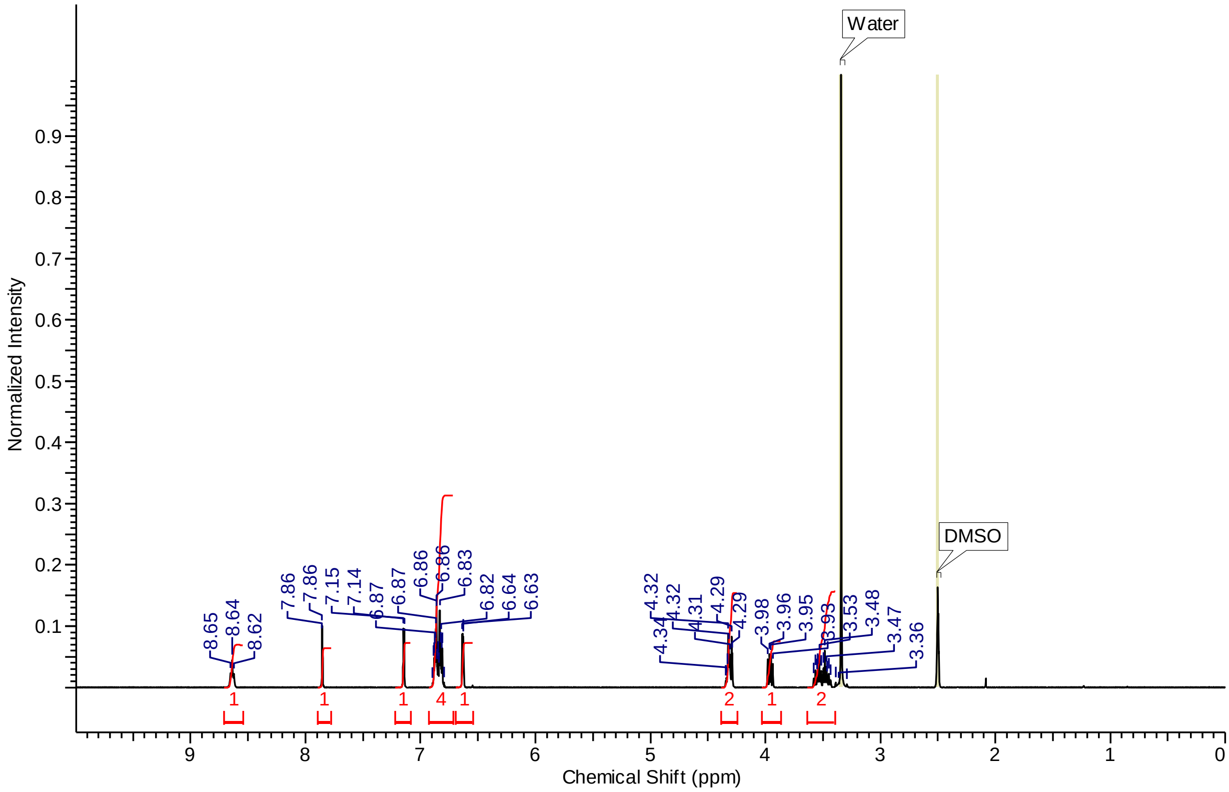
Water
DMSO
Normalized Intensity
Chemical Shift (ppm)

b

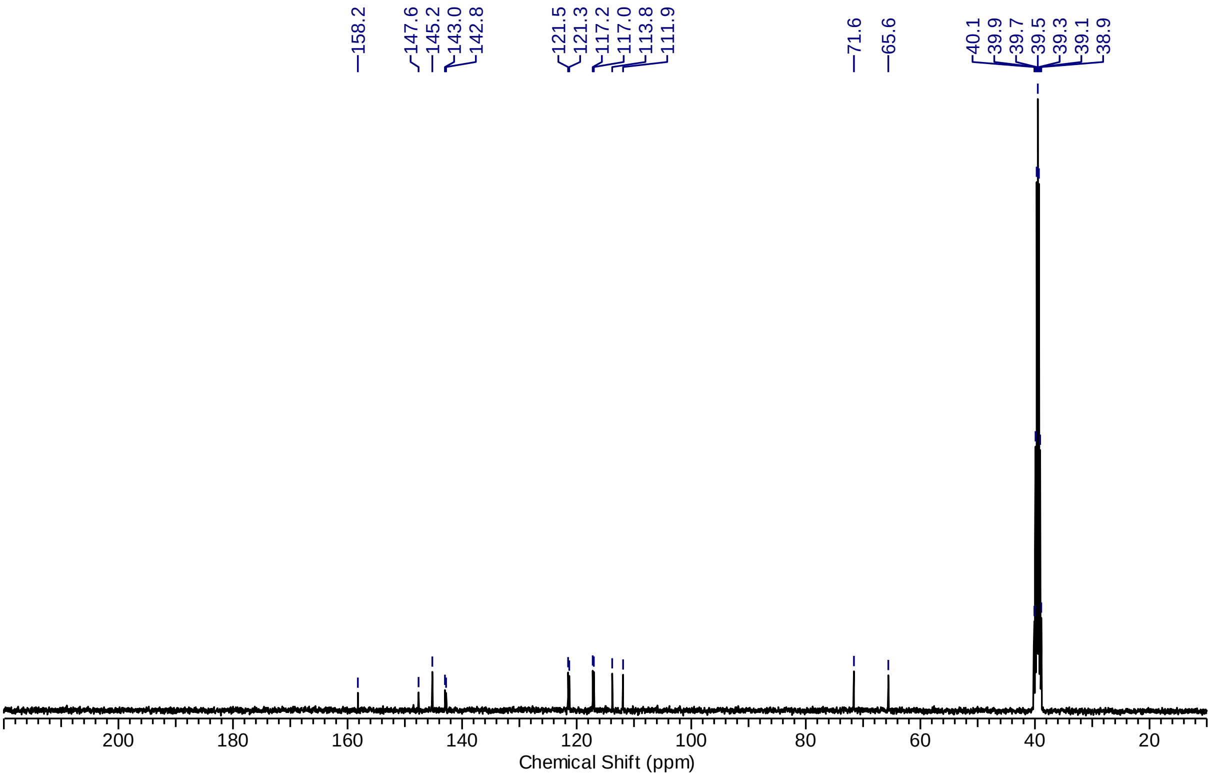
158.2
147.6
145.2
143.0
142.8
121.5
121.3
117.2
117.0
113.8
111.9
71.6
65.6
40.1
39.9
39.7
39.5
39.3
39.1
38.9
Chemical Shift (ppm)

c

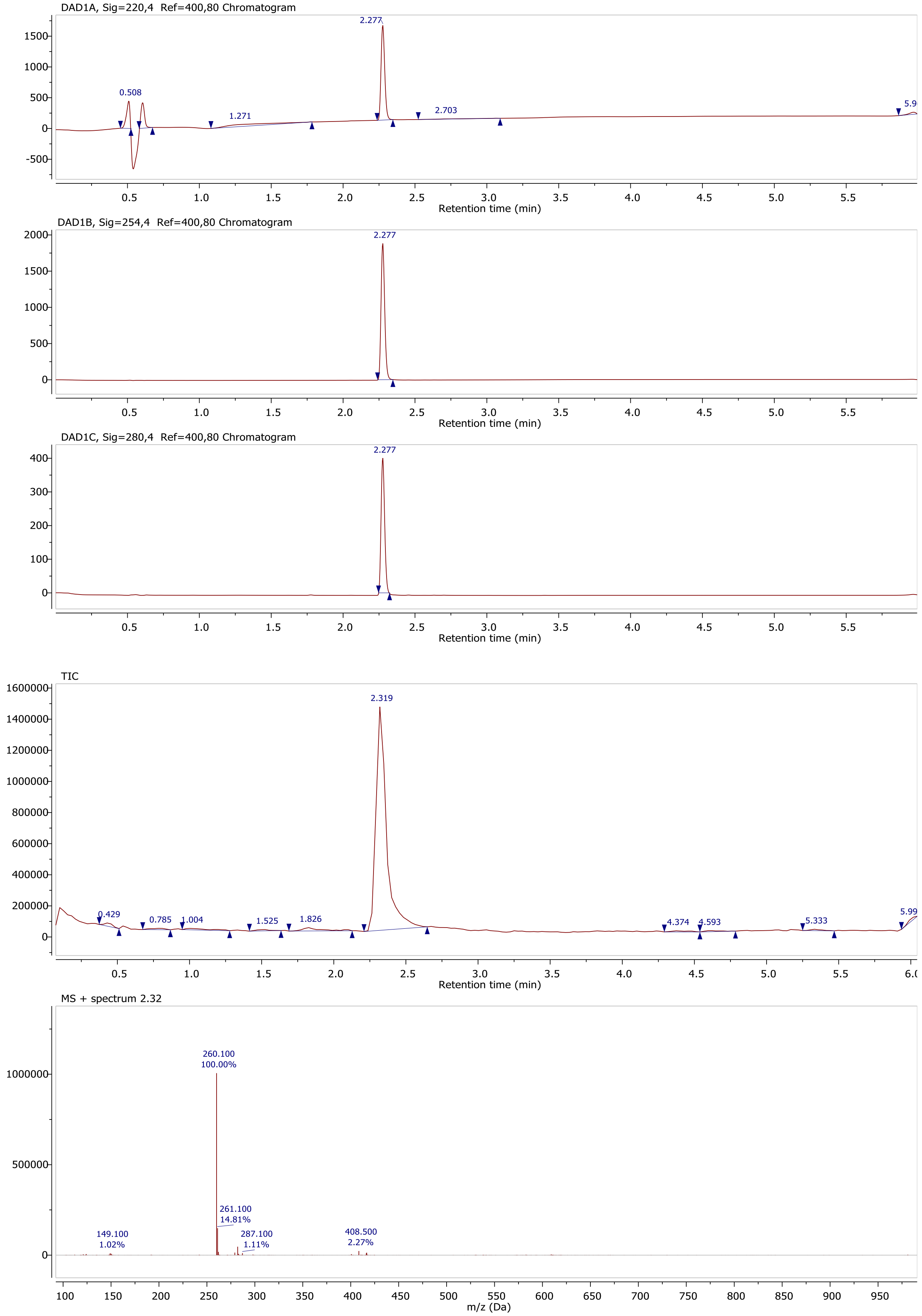
DAD1A, Sig=220,4 Ref=400,80 Chromatogram
0.508
1.271
2.277
2.703
5.9
Retention time (min)
DAD1B, Sig=254,4 Ref=400,80 Chromatogram
2.277
Retention time (min)
DAD1C, Sig=280,4 Ref=400,80 Chromatogram
2.277
Retention time (min)
TIC
2.319
0.429
0.785
1.004
1.525
1.826
4.374
4.593
5.333
5.99
Retention time (min)
MS + spectrum 2.32
260.100
100.00%
261.100
14.81%
149.100
1.02%
287.100
1.11%
408.500
2.27%
m/z (Da)

Supplementary Figure 11: ^{1}H NMR, ^{13}C NMR and LCMS of Abd-2b.
Data were recorded on Bruker Avance spectrometers (AVII400 or AVIII400) in the deuterated solvent stated. The field was locked by external referencing to the relevant deuteron resonance. Chemical shifts (δ) are reported in parts per million (ppm) referenced to the solvent peak. Panel a: ^{1}H NMR spectra. Panel b: ^{13}C NMR spectra. Panel c: LCMS.

a

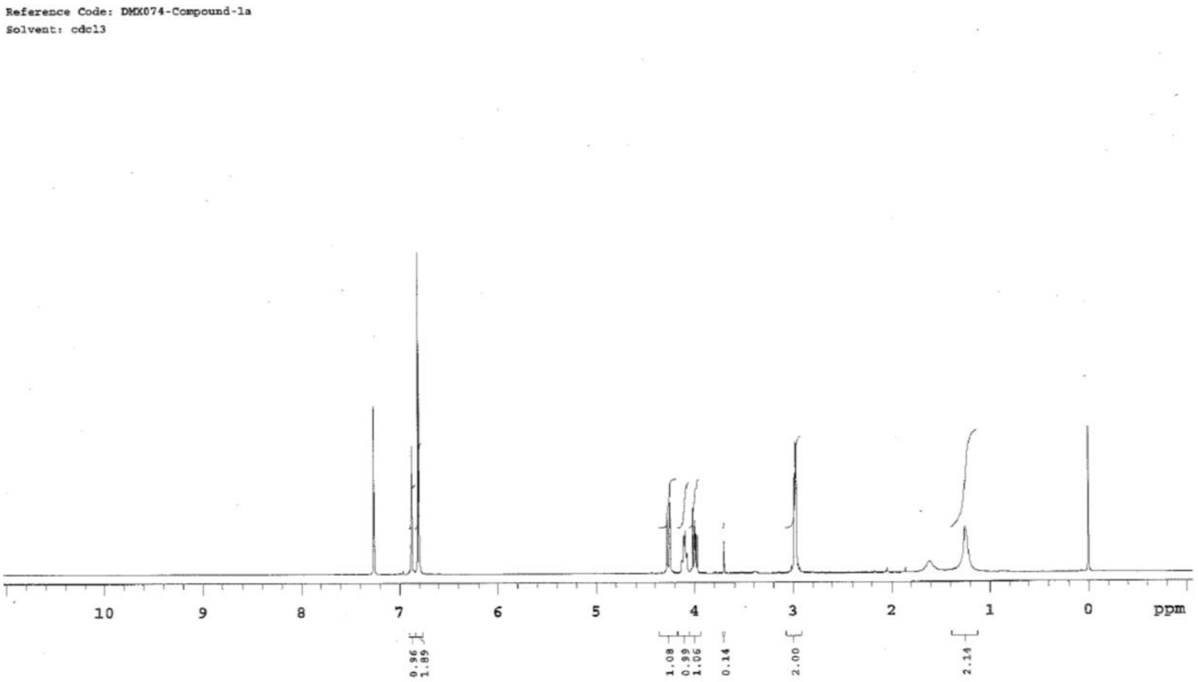
Reference Code: DMX074-Compound-1a
Solvent: cdcl3
10 9 8 7 6 5 4 3 2 1 0 ppm
0.96
1.89
1.08
0.99
1.06
0.14
2.00
2.14

b

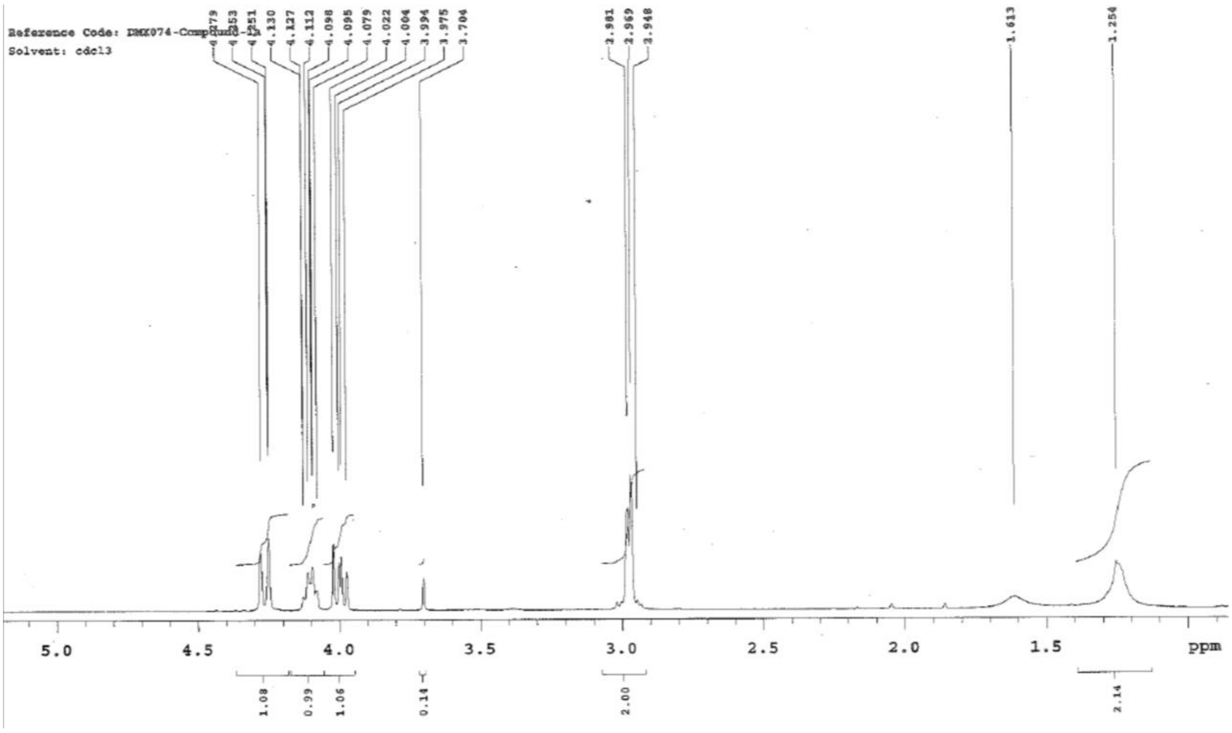
Reference Code: DMX074-Compound-1a
Solvent: cdcl3
4.279
4.253
4.251
4.130
4.127
4.112
4.098
4.095
4.079
4.022
4.004
3.994
3.975
3.704
2.981
2.969
2.948
1.613
1.254
5.0 4.5 4.0 3.5 3.0 2.5 2.0 1.5 ppm
1.08
0.99
1.06
0.14
2.00
2.14

c

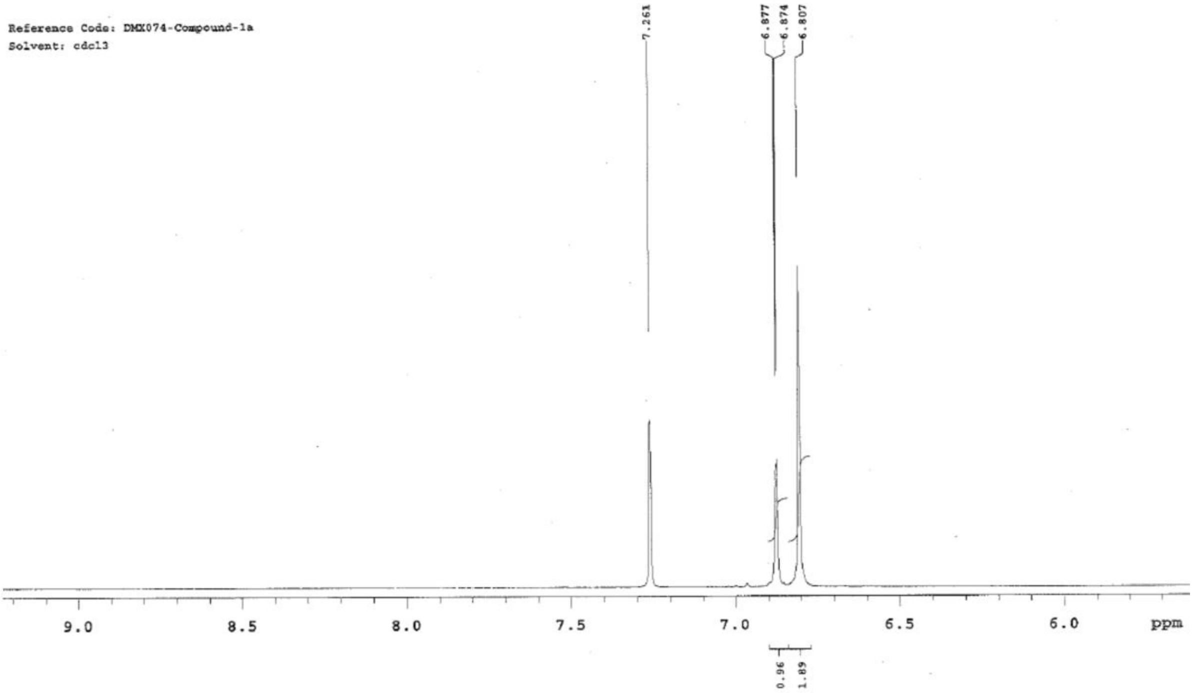

Supplementary Figure 12: ^{1}H NMR of Abd-3.

Data were recorded on Agilent MRDD2 (400 MHz) in the deuterated solvent stated. The field was locked by external referencing to the relevant deuteron resonance. Chemical shifts (δ) are reported in parts per million (ppm) referenced to the solvent peak. **Panel a**: Full ^{1}H NMR spectra. **Panel b**: ^{1}H NMR zoom for the aliphatic region. **Panel c**: ^{1}H NMR zoom for the aromatic region.

a

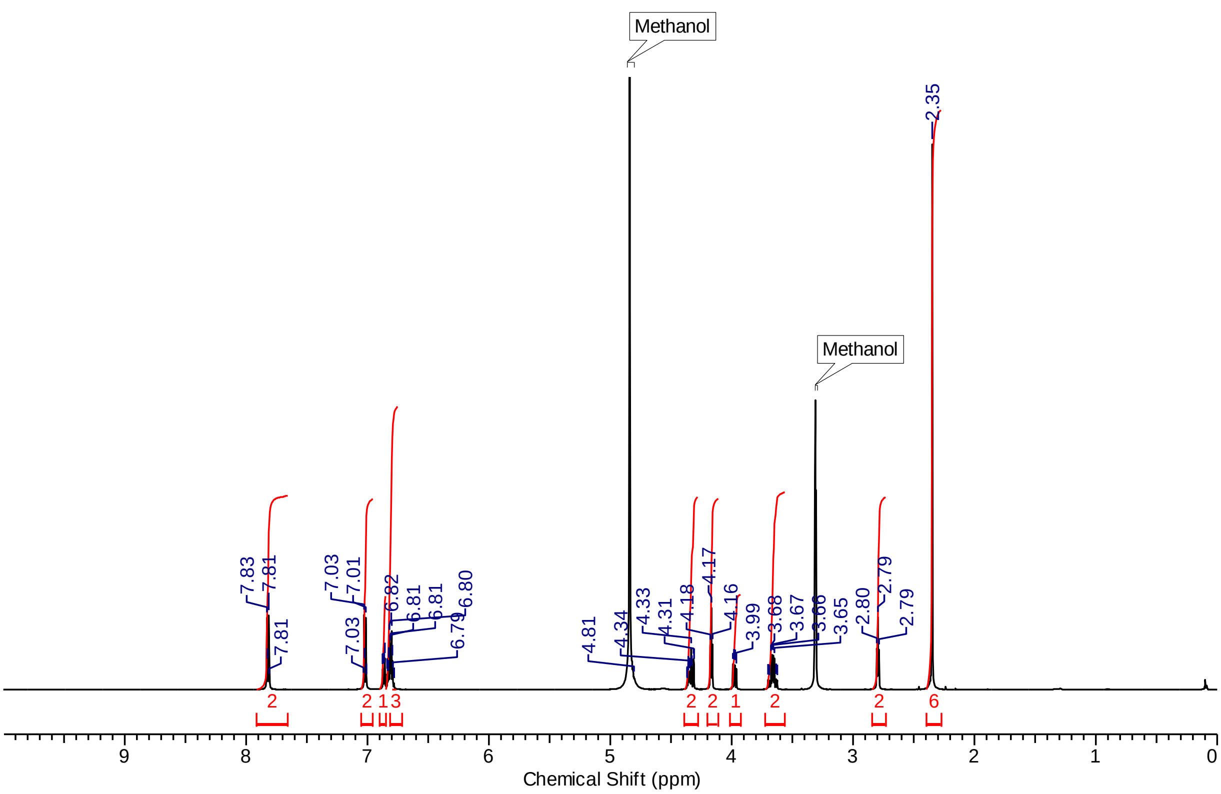

b

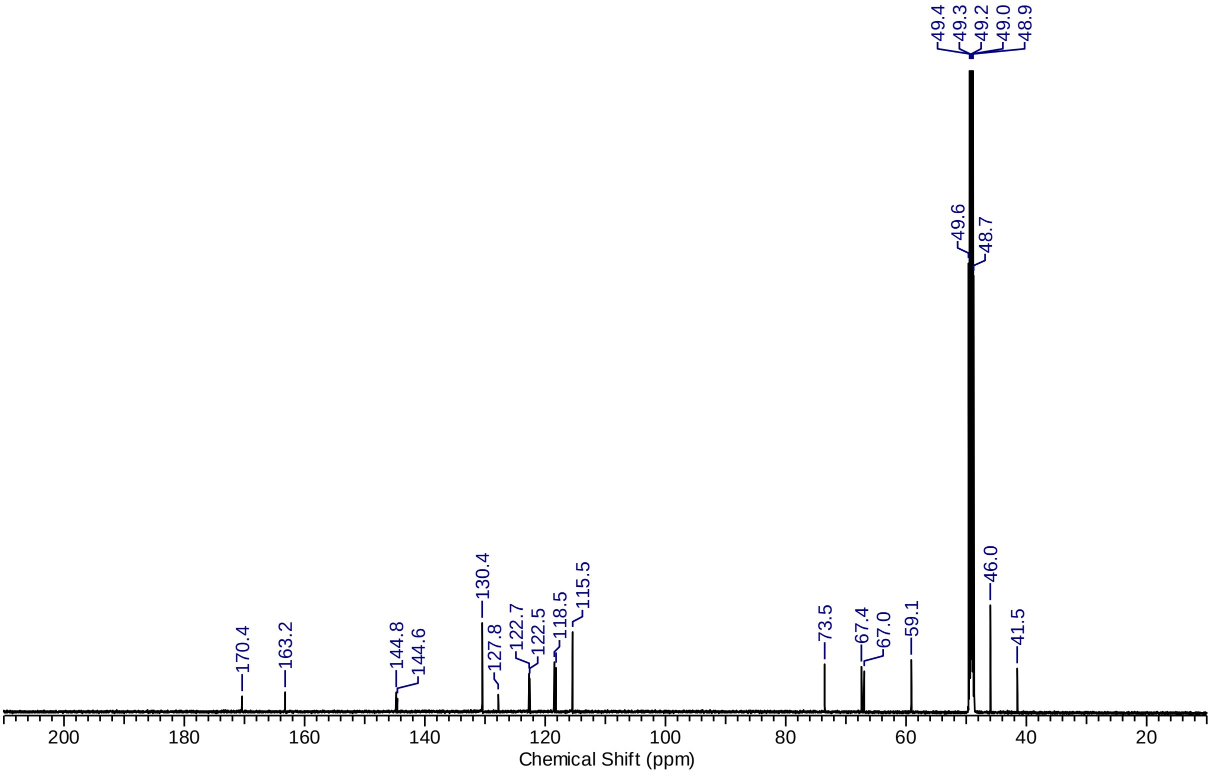

Supplementary Figure 13: ^{1}H NMR and ^{13}C NMR of Abd-4.

Data were recorded on Bruker Avance spectrometers (AVII400 or AVIII400) in the deuterated solvent stated. The field was locked by external referencing to the relevant deuteron resonance. Chemical shifts (δ) are reported in parts per million (ppm) referenced to the solvent peak. **Panel a**: ^{1}H NMR spectra. **Panel b**: ^{13}C NMR spectra.

a

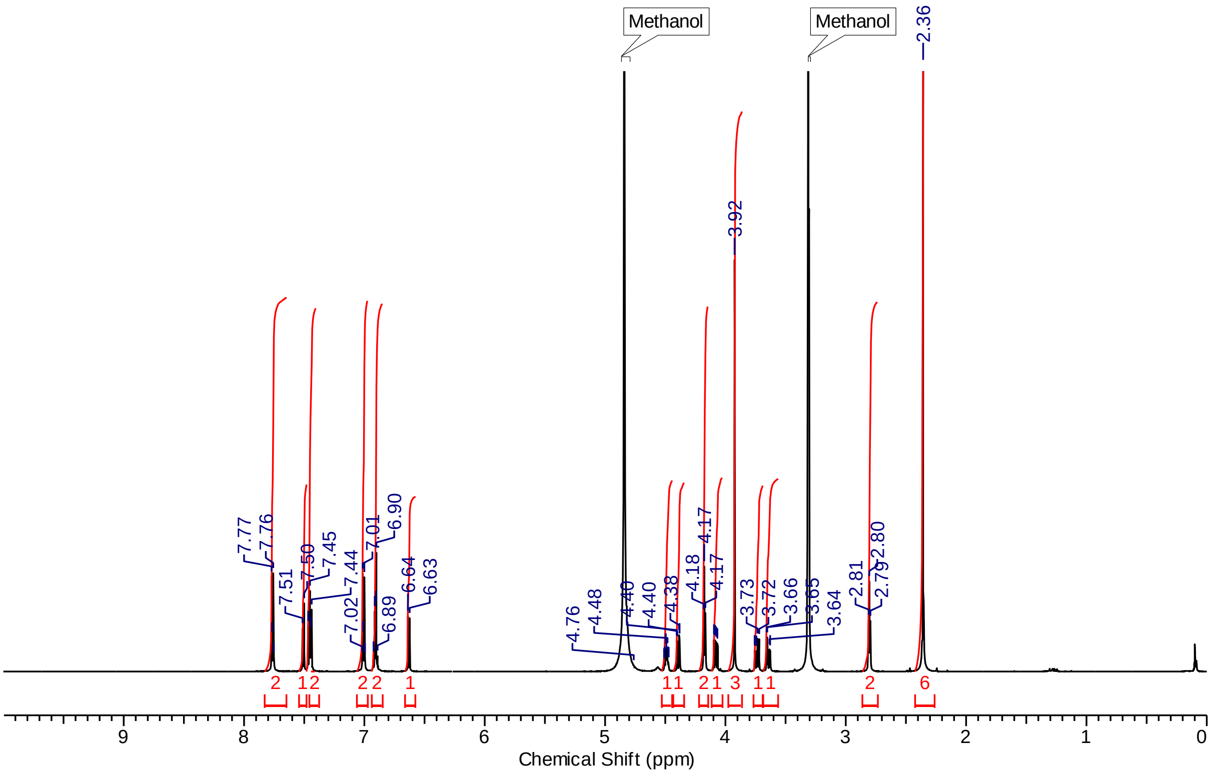

b

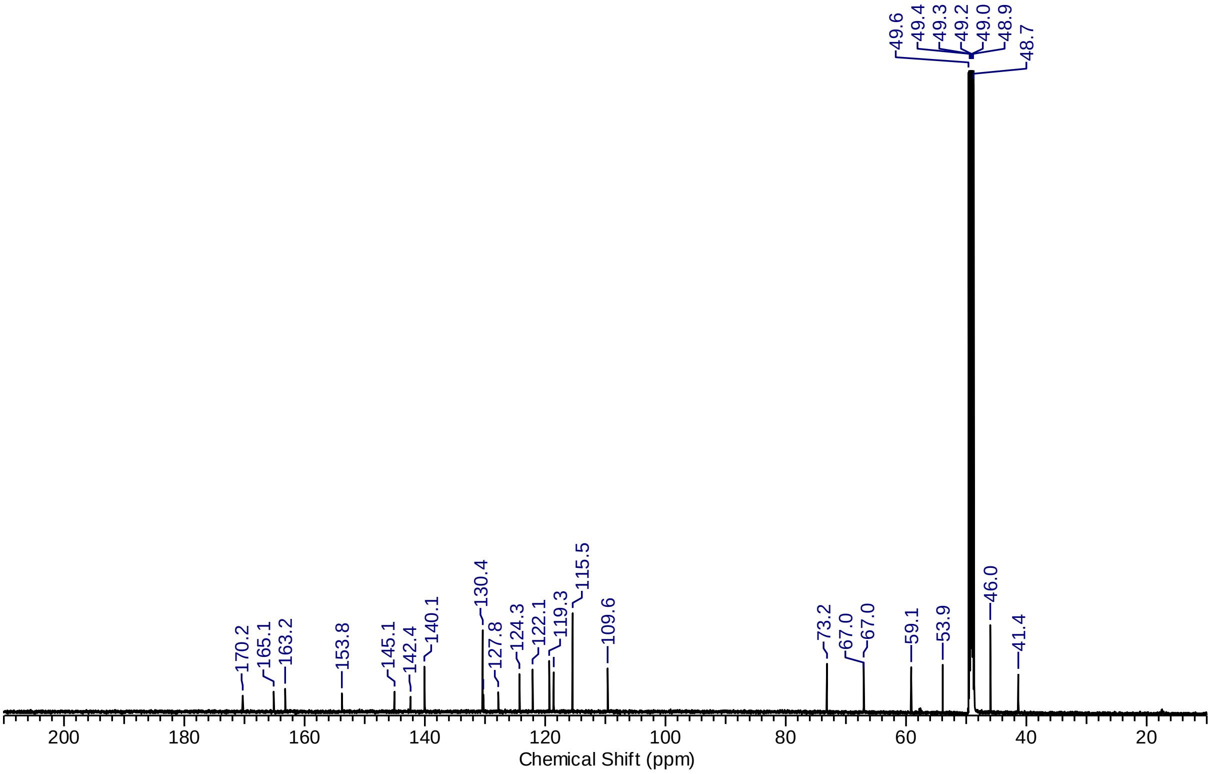

Supplementary Figure 14: ^{1}H NMR and ^{13}C NMR of Abd-5.

Data were recorded on Bruker Avance spectrometers (AVII400 or AVIII400) in the deuterated solvent stated. The field was locked by external referencing to the relevant deuteron resonance. Chemical shifts (δ) are reported in parts per million (ppm) referenced to the solvent peak. **Panel a**: ^{1}H NMR spectra. **Panel b**: ^{13}C NMR spectra.

a

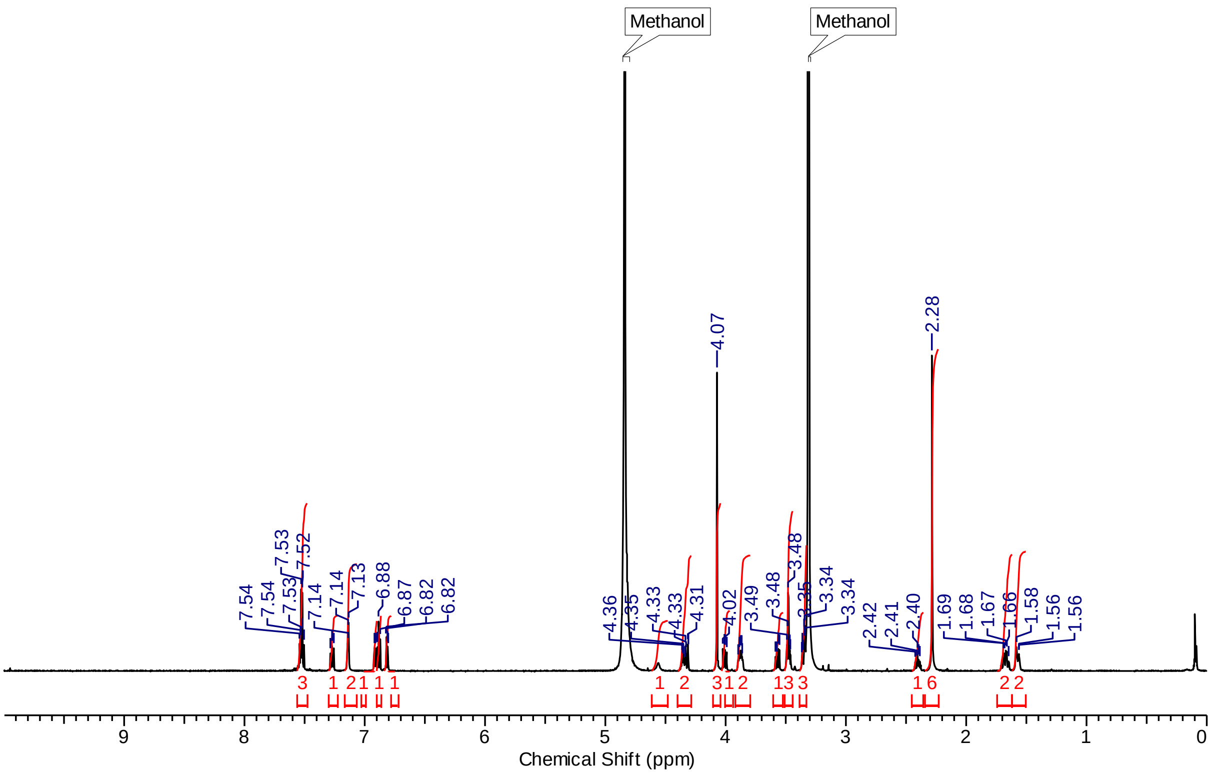

b

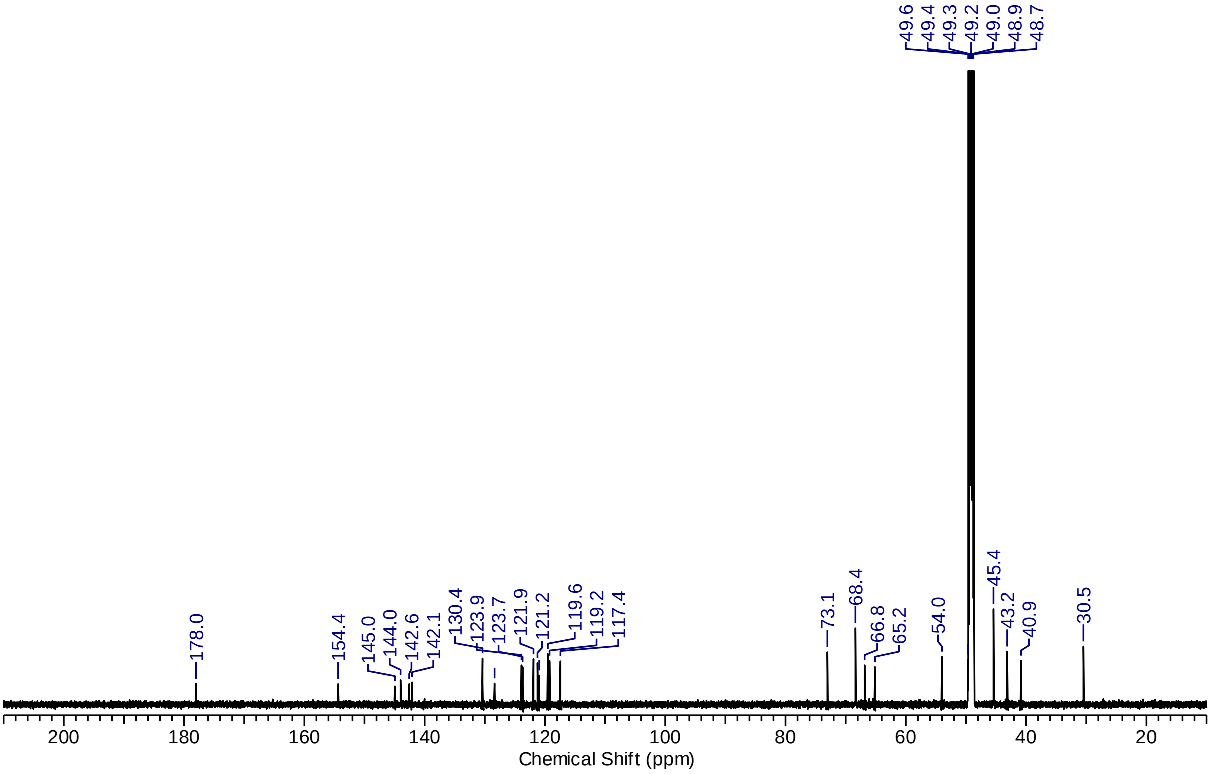

Supplementary Figure 15: ^{1}H NMR and ^{13}C NMR of Abd-6.

Data were recorded on Bruker Avance spectrometers (AVII400 or AVIII400) in the deuterated solvent stated. The field was locked by external referencing to the relevant deuteron resonance. Chemical shifts (δ) are reported in parts per million (ppm) referenced to the solvent peak. **Panel a**: ^{1}H NMR spectra. **Panel b**: ^{13}C NMR spectra.

a

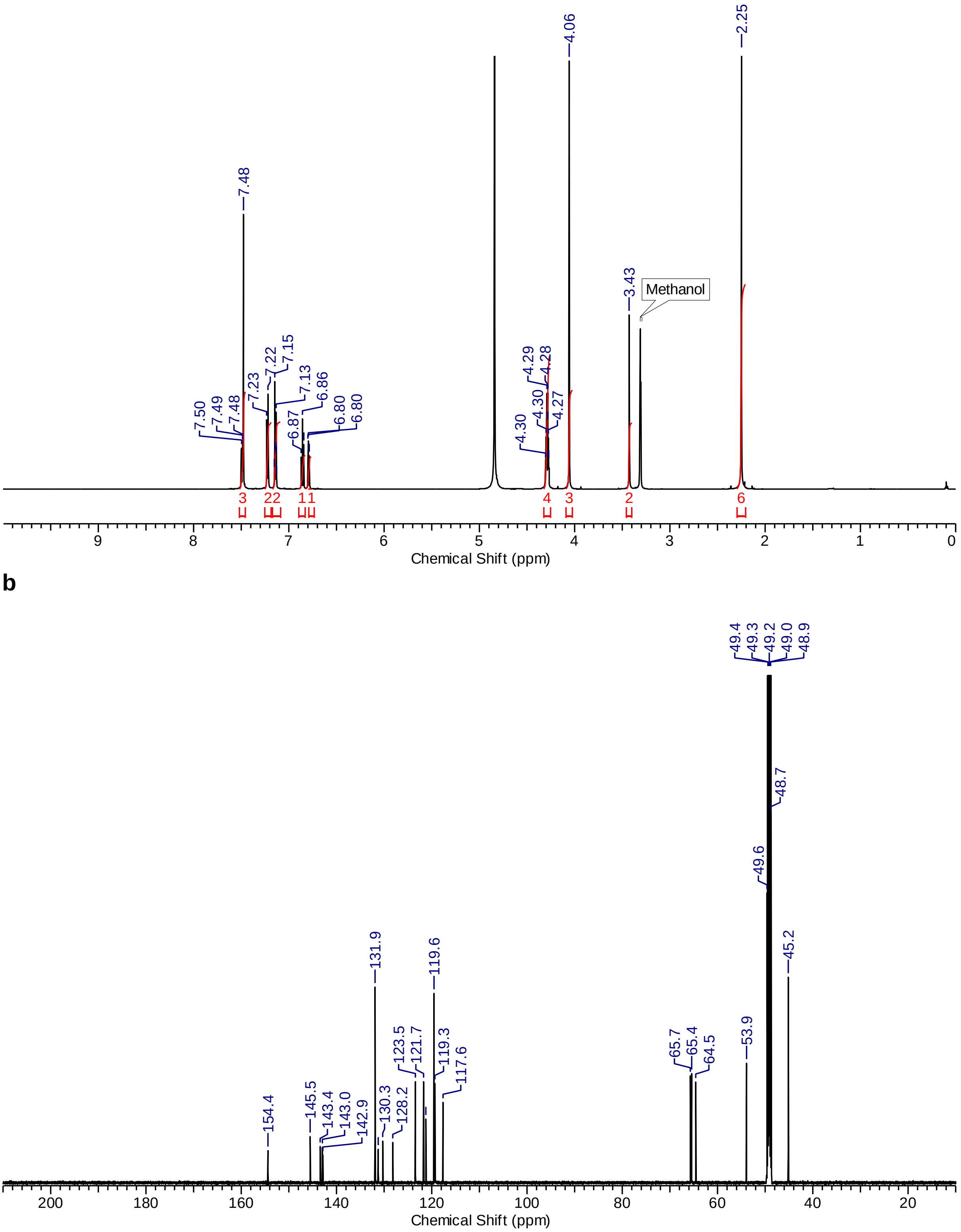

Supplementary Figure 16: ^{1}H NMR and ^{13}C NMR of Abd-7.

Data were recorded on Bruker Avance spectrometers (AVII400 or AVIII400) in the deuterated solvent stated. The field was locked by external referencing to the relevant deuteron resonance. Chemical shifts (δ) are reported in parts per million (ppm) referenced to the solvent peak. **Panel a**: ^{1}H NMR spectra. **Panel b**: ^{13}C NMR spectra.

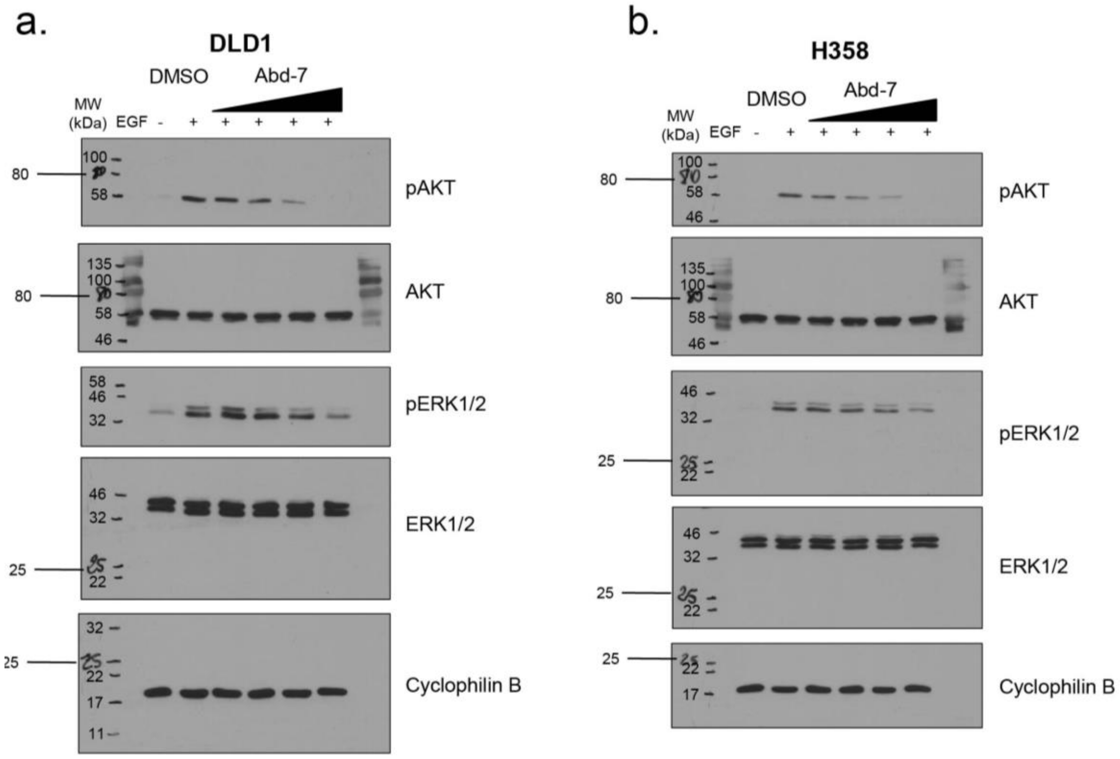

Supplementary Figure 17: Uncropped scans of western blots

(a) Full blots from figure 5a. (b) full blots from figure 5b. MW: Molecular Weight. EGF: Epidermal Growth Factor

a. Abd-2 Q61H

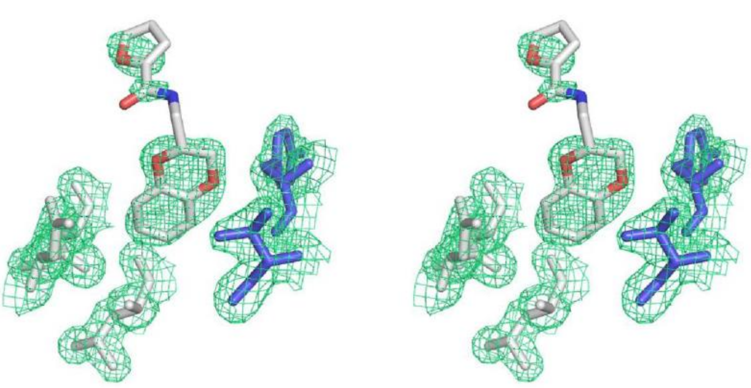

b. Abd-3 G12D

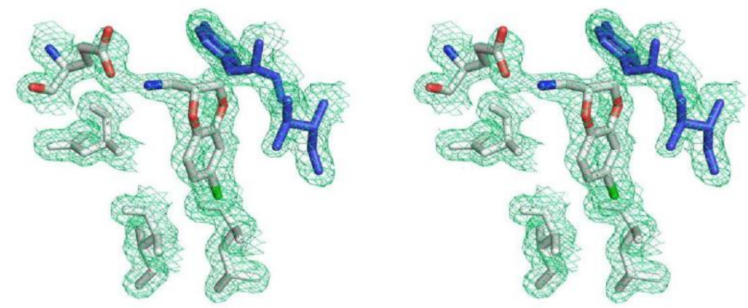

c. Abd-3 Q61H

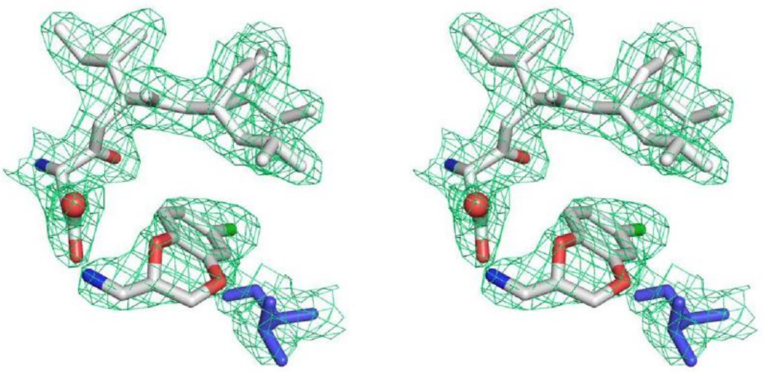

d. Abd-4 Q61H

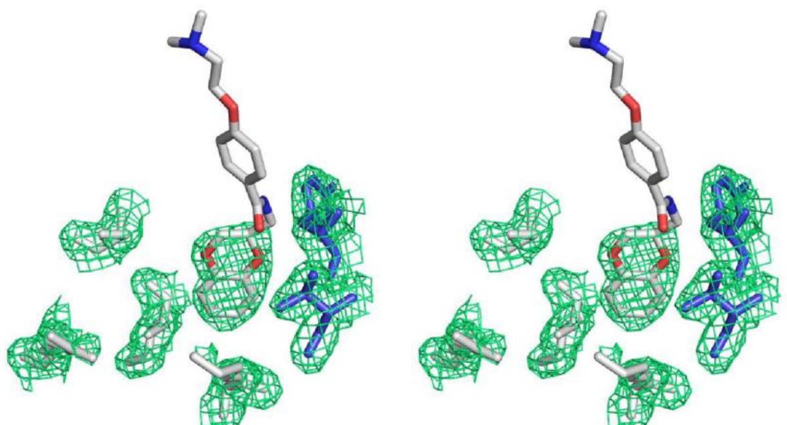

e. Abd-5 Q61H

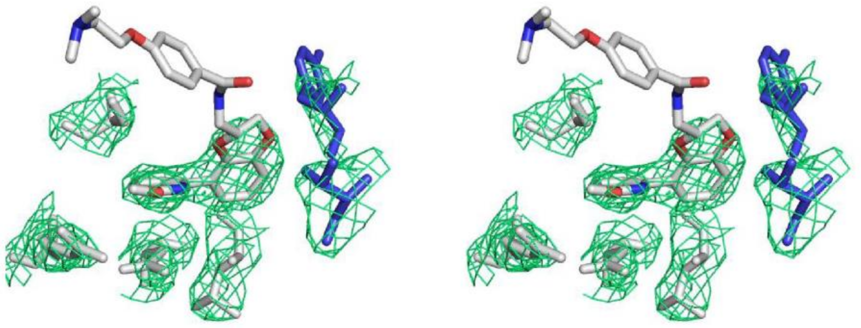

f. Abd-6 Q61H

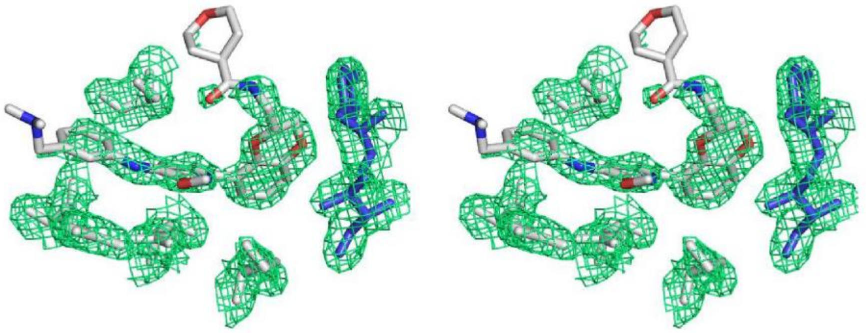

g. Abd-7 Q61H

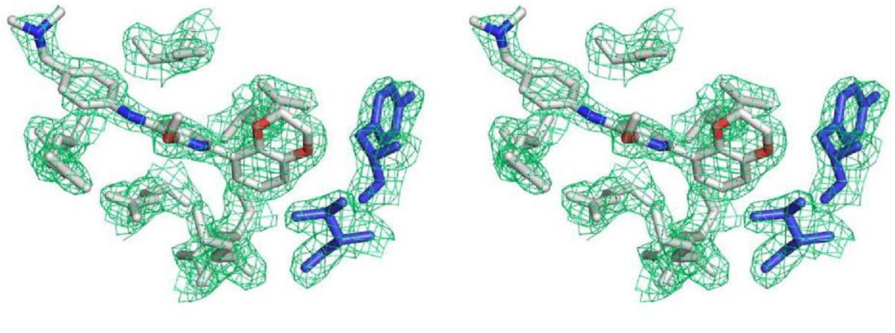

Supplementary Figure 18: Stereo images of Abd compounds

Wall-Eye stereo pictures with *2mFo-DFc* maps contoured at 1.0 r.m.s. of the different Abd-compounds and representative amino acids around the compound binding sites. (a) Abd-2 Q61H. (b) Abd-3 G12D. (c) Abd-3 Q61H. (d) Abd-4 Q61H. (e) Abd-5 Q61H. (f) Abd-5 Q61H. (f) Abd-6 Q61H. (g) Abd-7 Q61H.

Supplementary Table 1 Data Processing and Refinement Statistics for the $KRAS_{169}^{Q61H}$ GppNHp-Abd-2, Abd-3 and $KRAS_{188}^{G12D}$ Abd-3 structures.

	$KRAS_{169}^{Q61H}$-GPPNHP Abd-2	$KRAS_{169}^{Q61H}$-GPPNHP Abd-3	$KRAS_{188}^{G12D}$-GPPNHP Abd-3
**Data Collection**			
Space group	$P\ 2_12_12_1$	$P\ 2_12_12_1$	H3
Molecules/asymmetric unit	6	6	1
**Unit cell dimensions**			
a, b, c (Å)	63.60 ,118.76, 156.90	63.49, 118.45, 155.36	78.09, 78.09, 77.65
Resolution (Å)	59.38-1.66 (1.70- 1.66)	41.72-2.07 (2.12-2.07)	39.04-1.45 (1.5- 1.45)
Rmerge†	0.108 (2.229)	0.225 (1.750)	0.105 (0.802)
I/sigma	13.6(1.3)	12.2 (1.4)	8.2 (1.6)
Completeness (%)	100.0 (100.0)	99.9 (100.0)	100.0 (100.0)
Redundancy	13.4 (13.0)	12.9 (13.1)	5.0 (4.2)
**Refinement**			
Resolution (Å)	59.38- 1.66	41.72- 2.07	39.04- 1.45
No. of reflections	147744 (14611)	75652 (7452)	30994 (3108)
Rwork/Rfree	0.179/ 0.205	0.189/ 0.218	0.176/ 0.204
No. of atoms			
Protein	8258	8300	1384
Water	646	456	113
B factors			
Protein	36.8	43.8	25.6
Ligand	42.2	49.8	24.3
Water	37.9	44	31.9
R.m.s deviations			
Bond lengths (Å)	0.021	0.018	0.025
Bond angles (°)	2.1	1.9	2.21

Values in parentheses are for highest-resolution shell.

Supplementary Table 2. Data Processing and Refinement Statistics for the $KRAS_{169}^{Q61H}$ GppNHp-Abd-4, Abd-5, Abd-6 and Abd-7 structures.

	$KRAS_{169}^{Q61H-}$ GPPNHP Abd-4	$KRAS_{169}^{Q61H}$- GPPNHP Abd-5	$KRAS_{188}^{G12D}$- GPPNHP Abd-6	$KRAS_{188}^{G12D}$- GPPNHP Abd-7
**Data Collection**				
Space group	P $2_12_12_1$	P $2_12_12_1$	P $2_12_12_1$	P $2_12_12_1$
Molecules/asymmetric unit	6	6	6	6
**Unit cell dimensions**				
a, b, c (Å)	63.61, 118.60, 156.61	63.23, 117.21 156.45	63.39, 118.48, 156.28	156.12, 63.19, 117.73
Resolution (Å)	65.35-1.97(2.02-1.97)	49.17-2.6 (2.72-2.6)	63.39-1.82 (1.87 - 1.82)	65.06-2.02 (2.05 -2.02)
Rmerge†	0.170 (1.450)	0.058 (1.51)	0.106(1.380)	0.089 (0.589)
I/sigma	13.0(1.6)	19.1 (1.2)	15.5(1.5)	10.9 (2.7)
Completeness (%)	99.8 (97.7)	100 (100)	99.8(97.7)	95.0 (97.7)
Redundancy	12.9(9.3)	7.4 (7.4)	12.3(8.0)	4.7 (4.4)
**Refinement**				
Resolution (Å)	65.35-1.97	49.17-2.6	63.39-1.82	65.06-2.02
No. of reflections	84321 (5992)	36553 (2669)	111022 (10809)	73949 (7881)
Rwork/Rfree	18.1/20.7	20.3/23.3	18.4/20.7	20.0/23.3
No. of atoms				
Protein	8646	8146	8901	8696
Water	451	37	361	137
B factors				
Protein	39.8	65.9	38.7	38.2
Ligand	111.5	105	49.1	47.3
Water	39.6	39.8	39.5	30.4
R.m.s deviations				
Bond lengths (Å)	0.017	0.012	0.018	0.017
Bond angles (°)	1.94	1.67	1.95	1.93

Values in parentheses are for highest-resolution shell.

Supplementary Table 3. Assessment of potential interaction of Abd-7 with a human kinase panel

Kinase	% kinase activity in the presence of Abd-7
BRAF	104
CRAF	101
EGFR	93
MAPK1	126
MAPK2	111
MEK1	105
MEK2	101
mTOR	89
PDK1	107
PKBα	91
PKBβ	93
PKBγ	103
PI3KC2α	102
PI3KC2γ	108

A panel of 14 Kinases (relevant to the RAS pathway) has been screened against Abd-7 (10μM) and the kinase activity was measured. A <90% level of activity was observe for all the kinases screened, a clear indication that Abd-7 does not interfere with the kinase function of the selected and RAS relevant kinase proteins.

Supplementary References

1 McComsey, D. F. *et al.* Novel, broad-spectrum anticonvulsants containing a sulfamide group: pharmacological properties of (S)-N-[(6-chloro-2,3-dihydrobenzo[1,4]dioxin-2-yl)methyl]sulfamide (JNJ-26489112). *J Med Chem* **56**, 9019-9030, doi:10.1021/jm400894u (2013).

2 Takahashi, B. *et al.* Orally active ghrelin receptor inverse agonists and their actions on a rat obesity model. *Bioorg Med Chem* **23**, 4792-4803, doi:10.1016/j.bmc.2015.05.047 (2015).
